# Blood transcriptomic signatures predict poor outcomes in drug-susceptible pulmonary tuberculosis in Brazil

**DOI:** 10.1093/ajrccm/aamag156

**Published:** 2026-03-30

**Authors:** Simon C Mendelsohn, Bruno B Andrade, Mariana Araújo-Pereira, Gustavo C Amorim, Mzwandile Erasmus, Alice M S Andrade, Michelle Fisher, Marina C Figueiredo, Vanessa M Muwanga, Afranio L Kritski, Marcelo Cordeiro-Santos, Valeria C Rolla, Mark Hatherill, Timothy R Sterling, Thomas J Scriba, and members of the RePORT South Africa, Nicole Bilek, Yolundi Cloete, Katie Hadley, Rieyaat Hassiem, Lungisa Jaxa, Stanley Kimbung Mbandi, Faheemah Meyer, Humphrey Mulenga, Onke Nombida, Alison September, Ashley Veldsman, and RePORT-Brazil Consortia, Brenda Carvalho, Adriano Gomes, Cody Staats, Megan Turner

**Affiliations:** South African Tuberculosis Vaccine Initiative, Institute of Infectious Disease and Molecular Medicine and Division of Immunology, Department of Pathology, University of Cape Town, Cape Town, South Africa; Laboratório de Pesquisa Clínica e Translacional, Instituto Gonçalo Moniz, Fundação Oswaldo Cruz, Salvador, Bahia, Brazil; Laboratório de Pesquisa Clínica e Translacional, Instituto Gonçalo Moniz, Fundação Oswaldo Cruz, Salvador, Bahia, Brazil; Vanderbilt University Medical Center, Nashville, TN, United States; South African Tuberculosis Vaccine Initiative, Institute of Infectious Disease and Molecular Medicine and Division of Immunology, Department of Pathology, University of Cape Town, Cape Town, South Africa; Laboratório de Pesquisa Clínica e Translacional, Instituto Gonçalo Moniz, Fundação Oswaldo Cruz, Salvador, Bahia, Brazil; South African Tuberculosis Vaccine Initiative, Institute of Infectious Disease and Molecular Medicine and Division of Immunology, Department of Pathology, University of Cape Town, Cape Town, South Africa; Vanderbilt University Medical Center, Nashville, TN, United States; South African Tuberculosis Vaccine Initiative, Institute of Infectious Disease and Molecular Medicine and Division of Immunology, Department of Pathology, University of Cape Town, Cape Town, South Africa; Faculdade de Medicina, Universidade Federal do Rio de Janeiro, Rio de Janeiro, RJ, Brazil; Fundação de Medicina Tropical Doutor Heitor Vieira Dourado, Manaus, Amazonas, Brazil; Laboratorio de Pesquisa Clinica em Micobacterioses, Instituto Nacional de Infectologia Evandro Chagas, Fiocruz, Rio de Janeiro, Brazil; South African Tuberculosis Vaccine Initiative, Institute of Infectious Disease and Molecular Medicine and Division of Immunology, Department of Pathology, University of Cape Town, Cape Town, South Africa; Vanderbilt University Medical Center, Nashville, TN, United States; South African Tuberculosis Vaccine Initiative, Institute of Infectious Disease and Molecular Medicine and Division of Immunology, Department of Pathology, University of Cape Town, Cape Town, South Africa

**Keywords:** gene expression signature, prognosis, tuberculosis, biomarkers, treatment outcome

## Abstract

**Rationale:**

Non-sputum biomarkers to monitor tuberculosis treatment and predict poor outcomes are lacking.

**Objectives:**

To evaluate host-blood transcriptomic signatures for treatment monitoring and prognosis of death, treatment failure, and recurrence in adults with pulmonary tuberculosis.

**Methods:**

Adults with culture-confirmed, drug-susceptible pulmonary tuberculosis were enrolled at 5 Brazilian sites. Whole-blood PAXgene samples were collected at baseline, month 2 (M2), and end of treatment (EoT). Treatment failure was defined as sputum culture positivity at month 5 or later. Participants were followed for 24 months from treatment initiation for clinical or microbiological tuberculosis recurrence. Unfavorable outcomes were matched ∼1:3 to recurrence-free cure. Twenty-two published blood transcriptomic signatures were measured by microfluidic RT-qPCR and benchmarked against the WHO Target Product Profile (TPP) criteria.

**Measurements and Main results:**

We matched 263 participants with recurrence-free cure to 33 with treatment failure, 24 who died (tuberculosis/unknown cause), and 9 with recurrence. Signature scores generally declined from baseline to EoT. Multiple signatures measured at baseline and M2 predicted recurrence (AUC range 0.71-0.91), with waning performance when measured at EoT (AUC range 0.42-0.89). Against the WHO TPP, 2/22 signatures met minimum criteria at baseline, 13/22 at M2, and none at EoT. Prediction of treatment failure was poor across timepoints (AUC < 0.70). In contrast, several signatures measured at baseline predicted death during treatment or follow-up (AUC ≥ 0.80).

**Conclusions:**

Blood transcriptomic signatures tracked treatment response and predicted recurrence and death, meeting WHO TPP benchmarks at baseline and M2. These findings support prospective, biomarker-guided trials to individualize tuberculosis therapy—shortening regimens for early responders and intensifying care for high-risk patients.

Subject category: 11.6 Treatment of Tuberculosis or Latent Infection

At a Glance Commentary **Scientific Knowledge on the Subject: Non-sputum biomarkers for tuberculosis treatment monitoring and prognosis are lacking. Prior transcriptomic studies showed that blood signature scores decline during treatment and may reflect response to therapy, but most were limited by small sample sizes and few adverse outcomes, leaving uncertainty about their ability to predict recurrence, treatment failure, or death.** 

**What This Study Adds to the Field: In this large Brazilian cohort of adults with drug-susceptible pulmonary tuberculosis, several blood transcriptomic signatures tracked treatment response over time and predicted recurrence and death, but not treatment failure. Some signatures met WHO target product profile benchmarks at baseline or month 2, supporting their potential as non-sputum biomarkers for biomarker-guided treatment stratification and future prospective individualized tuberculosis treatment trials.** 

## Introduction

Despite effective antibiotics, tuberculosis (TB) treatment failure, recurrence, and death remain major challenges, especially in high-risk populations. In 2023, global treatment failure rates were estimated at 12% for new and relapse TB cases, increasing to 21% among people living with HIV (PLHIV), 19% in previously treated individuals (excluding relapse), and 29% among those with multidrug- or rifampicin-resistant TB.^1^ World Health Organization (WHO) guidelines for drug-susceptible pulmonary TB recommend sputum smear microscopy or culture at the end of the intensive phase to monitor treatment response.^2^ However, both have significant limitations.^3^ Culture is slow, and smear microscopy cannot distinguish viable from dead bacilli, or *Mycobacterium tuberculosis* (Mtb) from nontuberculous mycobacteria. Both methods rely on sputum, which is often unavailable—particularly in children, PLHIV, those with good response to treatment, and those with extrapulmonary or paucibacillary disease.^4^ Nucleic acid amplification tests, such as Xpert MTB/RIF, may remain positive despite effective therapy due to the persistence of DNA from dead mycobacteria. Consequently, treatment response is often assessed by non-specific and insensitive clinical measures, such as weight gain and symptom improvement. No validated non-sputum biomarkers currently exist in clinical practice to guide TB treatment decisions.

Standard regimens for drug-susceptible pulmonary TB, either the 6-month (2HRZE/4HR) or more recently, the 4-month rifapentine-moxifloxacin-containing regimen, are prescribed irrespective of disease severity.^2^ Some expert consensus guidelines recommend extending to a total of 9 months (2HRZE/7HR) in patients with cavitation on the initial chest radiograph and a positive culture at 2 months.^5^ These regimens include 4 potentially toxic drugs, which can cause adverse effects and contribute to poor adherence.^6^ However, clinical trials have shown that cure is possible in selected individuals with shorter regimens.^7^^,^^8^ To enable individualized treatment duration, rapid and accessible biomarkers—ideally from non-sputum samples like finger-prick blood—are needed to predict early cure or failure before treatment completion.

Peripheral blood transcriptomic signatures offer a promising non-sputum approach. Several host-response blood signatures have been associated with TB treatment response,^9-16^ including RISK6, which correlates with lung inflammation on PET-CT and inversely with sputum mycobacterial load.^13^ The prognostic Zak16 transcriptomic signature tracked disease resolution and identified patients at risk of failure within 4 weeks of starting treatment.^12^ These findings have been replicated in HIV-negative and HIV-positive populations across South Africa, Brazil, and a multi-country study in Bangladesh, Georgia, Lebanon, and Madagascar.^13^^,^^17^ In comparative studies, transcriptomic signatures outperformed traditional biomarkers like C-reactive protein.^18^ In the Catalysis cohort, RISK6, Zak16, and Sweeney3 signatures distinguished TB patients with cure from those with treatment failure as early as the point of diagnosis and with high accuracy at treatment end.^12-14^ Despite promising results, most studies validating transcriptomic signatures have been limited by small sample sizes and few adverse outcomes. To our knowledge, no large prospective cohort has evaluated the performance of multiple RT-qPCR-based transcriptomic signatures for predicting TB treatment failure, recurrence, or death.

Accurate, non-sputum biomarkers are critically needed to monitor TB treatment response and guide therapy modification. Transcriptomic signatures could support regimen shortening in early responders, intensification for slow responders, and continuation for those at risk of relapse. Such signatures may also be used to expedite drug development and treatment trials.^18^ Host-blood transcriptomic signatures are now feasible for point-of-care implementation.^19-22^ We evaluated parsimonious host-response blood transcriptomic signatures to monitor TB treatment and predict adverse outcomes in adults with drug-susceptible pulmonary TB.

## Methods

### Study design and participants

Regional Prospective Observational Research for Tuberculosis (RePORT)-Brazil is a prospective observational cohort study that enrolled participants at 5 centers: 3 in the state of Rio de Janeiro (City of Rio de Janeiro: (1) Instituto Nacional de Infectologia and (2) Clínica da Família Rinaldo Delamare. Duque de Caxias: (3) Duque de Caxias Health Center), one in Salvador (Instituto Brasileiro para Investigação da Tuberculose), and one in Manaus (Fundação Medicina Tropical Dr. Heitor Vieira Dourado). Details of the study cohort have been published previously.^23^ This was a case-control study nested within the observational cohort. For this analysis, adults (≥18 years) initiating treatment for culture-confirmed drug-susceptible pulmonary TB were included. Sputum was solicited at month 5 of treatment for culture, and TB treatment failure was defined as a culture positive for Mtb. Cause of death during treatment or study follow-up was adjudicated by site investigators and classified as: (1) deaths directly attributable to TB; (2) deaths from unknown causes (possibly related to TB or with TB as a contributing factor); and (3) deaths from unrelated causes (eg, trauma, non-natural causes). Deaths from unrelated causes were excluded from analyses. TB recurrence was evaluated over 24 months following treatment initiation through symptom-triggered investigations (eg, ≥2 weeks of cough, fever, night sweats, or weight loss, or any hemoptysis), which included chest radiography and sputum smear and culture testing. Microbiologically confirmed recurrence required at least one sputum specimen culture-positive for Mtb. Clinical recurrence, determined by site investigators, was defined by compatible signs and symptoms of TB, with or without histopathology suggestive of TB (eg, necrotizing or caseating granulomas). Individuals with treatment failure or recurrent TB were treated or re-treated according to local guidelines.

### Blood sample collection and RNA extraction

Venous blood (2.5 mL) was collected in PAXgene RNA tubes (PreAnalytiX, Switzerland) at baseline, month 2, and at the end of TB treatment (EoT) for all participants, frozen at −20 °C, and shipped to the Instituto Gonçalo Moniz, Fundação Oswaldo Cruz in Salvador, Brazil. Additional PAXgene samples were collected at recurrence visits in participants with TB recurrence. Samples were thawed, and RNA was manually extracted using the PAXgene Blood RNA Kit (Qiagen, Germany) according to the manufacturer’s instructions. Approximately 50 ng of RNA (∼8.3 ng/µL in 6 µL water) per sample were plated into 96-well PCR plates, frozen at −80 °C, and shipped to the South African Tuberculosis Vaccine Initiative (SATVI) in Cape Town, South Africa.

### Sample size and matching

All available RNA samples from individuals with unfavorable outcomes (treatment failure, death, or TB recurrence) were matched to participants who completed follow-up with recurrence-free cure. Participants who were lost to follow-up or withdrawn were excluded from the matching procedure. Propensity (or risk) scores^24^ were estimated using logistic regression (Supplementary Methods).

### Transcriptomic signature panel design and measurement

Published TB transcriptomic signatures were selected based on diagnostic and prognostic performance reported in recent systematic reviews and head-to-head comparisons,^25-29^ as well as ongoing work at the SATVI laboratory.^30^ Most of the included signatures map to interferon (IFN) response and inflammatory gene pathways. We included 22 parsimonious signatures—defined as those derived using feature reduction during discovery—based on: (1) number of transcripts (<30); (2) availability of primer-probe sequences or predesigned TaqMan assays; and (3) evaluation in at least one external validation cohort (Table 1). Signatures were translated to a microfluidic, multiplex real-time quantitative PCR (RT-qPCR) platform, reparameterized, and measured as previously described (Supplementary Methods), using a panel of pre-qualified TaqMan gene expression primer-probe assays (Table S1).^30-32^

**Table 1:** Characteristics of host blood transcriptomic signatures included in the analysis.

Signature^a^	Reference	Signature model	Discovery cohort country	Discovery cohort age group	Discovery cohort application
**da Costa3**	*Tuberculosis* (2015)^43^	Random forest	Brazil	Adults	Diagnostic; TB vs. LTBI, HC, and OD
**de Araujo1 (NPC2)**	*Front Microbiol* (2016)^44^	Standardized expression	Brazil	Adults	Diagnostic; TB vs. LTBI and HC
**Duffy5**	*J Infect Dis* (2022)^37^	Mean standardized expression	Mice with contained Mtb infection		Predict TB disease and containment of Mtb infection
**Duffy9 (10)** ^b^	*PLoS One* (2019)^45^	Six-class multinomial random forest	South Africa, Malawi	Adults	Diagnostic, TB vs. LTBI and OD
**Francisco2**	*J Infect* (2017)^46^	Random forest	China	Adults	Diagnostic; TB vs. OD and HC
**Gjøen7**	*Sci Rep* (2017)^47^	LASSO regression	India	Children	Diagnostic; TB vs. HC
**Gliddon3**	*Front Immunol* (2021)^48^	_DeltaCT_ZNF296 – (_DeltaCT_FCGR1A + _DeltaCT_C1QB)	South Africa, Malawi	Adults	Diagnostic; TB vs. LTBI
**Gliddon4**	*Front Immunol* (2021)^48^	(_DeltaCT_TMCC1 + _DeltaCT_PRDM1) – (_DeltaCT_GBP6 + _DeltaCT_ARG1)^c^	South Africa, Malawi	Adults	Diagnostic; TB vs. OD
**Jacobsen3**	*J Mol Med* (2007)^49^	Linear discriminant analysis	Germany	Adults	Diagnostic; TB vs. LTBI and HC
**Kaforou22 (27)** ^d^	*PLoS Med* (2013)^50^	Disease risk score	South Africa, Malawi	Adults	Diagnostic; TB vs. LTBI
**Maertzdorf4**	*EMBO Mol Med* (2016)^51^	_RawCT_ID3−[(_RawCT_GBP1 + _RawCT_IFITM3 + _RawCT_P2RY14)/3] (adapted from Suliman et al.^52^)	India	Adults	Diagnostic; TB vs. HC and LTBI
**Penn-Nicholson6**	*Sci Rep* (2020)^13^	Pair-wise ensemble structure	South Africa	Adolescents	Prognostic; TB progressors vs. non-progressors
**Rajan5**	*Clin Infect Dis* (2019)^53^	Unsigned sums	Uganda	Adults	Diagnostic; HIV + TB vs. HIV + HC
**Roe1 (BATF2)**	*JCI Insight* (2016)^54^	Standardized expression	United Kingdom	Adults	Diagnostic (TB vs. HC and TB patients 2-4 years post recovery)
**Roe3**	*Clin Infect Dis* (2020)^55^	SVM (linear kernel)	United Kingdom	Adults	Prognostic; TB progressors vs. non-progressors
**Sambarey10**	*EBioMedicine* (2017)^56^	Linear discriminant analysis	India	Adults	Diagnostic; TB vs. HC and LTBI
**Satproedprai7**	*Genes Immun* (2015)^57^	LASSO regression	Thailand	Adults	Diagnostic; TB vs. HC and previous TB patients
**Suliman2**	*Am J Respir Crit Care Med* (2018)^52^	Pair-wise ensemble structure	The Gambia, South Africa	Adults	Prognostic; TB progressors vs. non-progressors
**Suliman4**					
**Sweeney3**	*Lancet Respir Med* (2016)^58^; *JAMA Netw Open* (2018)^14^	_RawCT_KLF2 – (_RawCT_GBP5 + _RawCT_DUSP3)/2	France, Malawi, South Africa, United Kingdom, USA	Adults	Diagnostic; TB vs. HC + LTBI and OD
**Thompson5**	*Tuberculosis* (2017)^12^	Pair-wise ensemble structure	South Africa	Adults	Monitoring TB treatment response
**Xpert MTB Host Response (Xpert HR)**	*Lancet Glob Health* (2024)^22^	(_DeltaCT_GBP5 + _DeltaCT_DUSP3)/2	See Sweeney3 signature		

a Signatures are named by first author and number of transcripts included in the model (eg, Author11). Numbers in brackets indicate the original number of transcripts in the published model. Some signatures have a reduced number of transcripts when translated to RT-qPCR due to duplicate transcript symbols (IDs), high-primer probe failure rate (poor amplification efficiency), or where transcript sequences from an original discovery cohorts could not be mapped to a more recent reference transcriptome.

b The CERKL1 primer-probe assay failed during panel optimization and was removed from the Duffy10 signature.

c See Signature reparameterization in Supplemental Methods.

d Three transcripts (C1QB, C1QC, and GBP6) were excluded from the Kaforou27 signature due to a high primer-probe assay failure rate. Unique primer-probe assays could not be designed for both of the FCGR1B transcript variants; only one FCGR1B primer-probe was included in the Kaforou22 model. We were unable to design a primer-probe for LOC728744; all information had been withdrawn from both the NCBI and RefSeq databases.

Abbreviations: HC, healthy controls; LASSO, Least Absolute Shrinkage and Selection Operator; LTBI, latent tuberculosis infection; OD, other diseases; SVM, support vector machines.

### Statistical analysis

All analyses were performed using R Statistical Software version 4.2.1 (R Foundation for Statistical Computing, Vienna, Austria). Signature score distributions were summarized using the median and interquartile range (IQR). Differences between 2 groups were assessed using the Wilcoxon Signed-Rank test for paired data and the Mann–Whitney U test for unpaired data. For comparisons across 3 or more groups, the Kruskal-Wallis test was used for unpaired data and the Friedman test for paired data. Spearman’s rank correlation was used to assess relationships between signature scores. Only samples with complete assay data were included in the correlation matrices. A 2-sided alpha of <0.05 was considered statistically significant.

To evaluate prognostic performance, the area under the receiver operating characteristic (ROC) curve (AUC) was calculated using the *pROC* package,^33^ and 95% confidence intervals (CIs) were computed using the DeLong method.^34^ AUCs were calculated for each candidate signature to distinguish cure from treatment failure, recurrence, or death at each treatment timepoint. Sensitivity, specificity, positive predictive value (PPV), and negative predictive value (NPV) were calculated using binary outcome indicators and standard formulae and benchmarked against the WHO Target Product Profile (TPP) for TB treatment monitoring and optimization (Table S3) at baseline/diagnosis (sensitivity 90%, specificity 70%), during (sensitivity 75%, specificity 80%), and at the end (sensitivity 80%, specificity 90%) of TB treatment.^35^^,^^36^ The number needed to treat (NNT) was calculated as 1/PPV. Wilson score intervals were used to calculate 95% CIs for performance metrics.

### Ethical approval

The study protocol was approved by the ethics committee of the Maternidade Climério de Oliveira in Salvador, Brazil; the institutional research ethics committees at each participating site; and Vanderbilt University Medical Center. The University of Cape Town Faculty of Health Sciences Human Research Ethics Committee (HREC 594/2018) approved the analysis of transcriptomic signatures on all samples collected. All participants provided written informed consent.

### Role of funding sources

The funders of this study had no role in study design, data collection, analysis, interpretation, or writing of the manuscript.

## Results

### Recruitment of TB patients and matching

**Figure 1:**
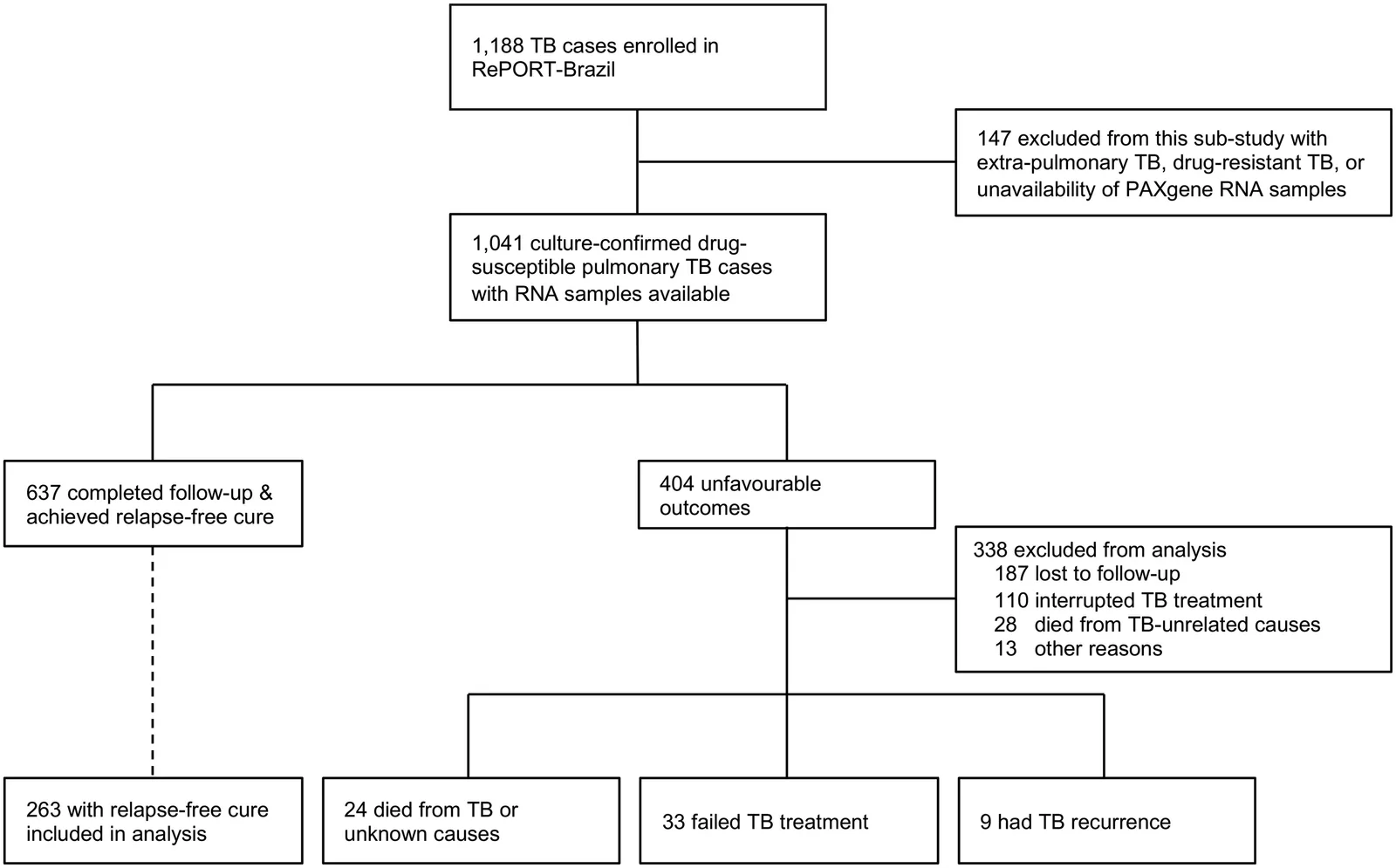
Study flow diagram.

Between June 2015 and June 2019, 1188 TB patients were enrolled in the RePORT-Brazil cohort (Figure 1), of whom 1041 with culture-confirmed drug-susceptible pulmonary TB had baseline RNA samples available. Of the 1041 participants eligible for this sub-study, 637 (61.2%) completed follow-up with a recurrence-free cure; 297 (28.5%) did not complete treatment or were lost to follow-up; 28 (2.7%) died from TB-unrelated causes; and 13 (1.2%) discontinued follow-up for other reasons. Overall, 66 (6.3%) participants with an unfavorable treatment outcome were included in the analysis: 33 (3.2%) who failed treatment, 24 (2.3%) who died from TB-related or unknown causes during treatment or study follow-up, and 9 (0.9%) who had TB recurrence. Of the 9 recurrence cases, 3 were microbiologically confirmed and 6 were clinically diagnosed.

All participants with an unfavorable outcome (treatment failure, death, or recurrence) were matched to 263 participants with a recurrence-free cure (Figure 1). This included 173 participants selected in both matching procedures, 42 in the individual-endpoint matching only, and 48 in the combined-endpoint matching only. Their characteristics are displayed in Table 2, stratified by outcomes status.

**Table 2:** Characteristics of the study population.

Characteristic	**Death due to TB or unknown cause, *N*** = **24**	**TB treatment failure, *N*** = **33**	**TB recurrence, *N*** = **9**	**Combined unfavorable outcomes, *N*** = **66**	**Recurrence-free cure, *N*** = **263**
**Male, *n* (%)**	18 (75.0)	17 (51.5)	7 (77.8)	42 (63.6)	172 (65.4)
**Median age, years (IQR)**	42.0 (32.8-47.5)	34.0 (25.0-51.0)	38.0 (34.0-40.0)	38.5 (27.2-49.8)	39.0 (26.0-53.0)
**City, *n* (%)**					
** Manaus**	19 (79.2)	18 (54.5)	5 (55.6)	42 (63.6)	104 (39.5)
** Rio de Janeiro**	2 (8.3)	5 (15.2)	4 (44.4)	11 (16.7)	98 (37.3)
** Salvador**	3 (12.5)	10 (30.3)	0 (0.0)	13 (19.7)	61 (23.2)
**Race, *n* (%)**					
** Brown**	17 (70.8)	16 (48.5)	6 (66.7)	39 (59.1)	152 (57.8)
** White**	2 (8.3)	5 (15.2)	1 (11.1)	8 (12.1)	58 (22.1)
** Black**	3 (12.5)	9 (27.3)	2 (22.2)	14 (21.2)	49 (18.6)
** Indian**	2 (8.3)	3 (9.1)	0 (0.0)	5 (7.6)	2 (0.8)
** Asian**	0 (0.0)	0 (0.0)	0 (0.0)	0 (0.0)	1 (0.4)
** Unknown/not reported**	0 (0.0)	0 (0.0)	0 (0.0)	0 (0.0)	1 (0.4)
**Smoking History, *n* (%)**					
** Never**	7 (29.2)	18 (54.5)	1 (11.1)	26 (39.4)	138 (52.5)
** Former**	14 (58.3)	8 (24.2)	5 (55.6)	27 (40.9)	84 (31.9)
** Current**	3 (12.5)	7 (21.2)	3 (33.3)	13 (19.7)	41 (15.6)
**Median BMI, kg/m^2^ (IQR)**	18.3 (16.8-20.7)	21.3 (17.9-23.0)	21.0 (19.9-22.2)	20.1 (17.3-22.8)	20.6 (18.8-22.9)
**Living with HIV, *n* (%)**	17 (70.8)	5 (15.2)	6 (66.7)	28 (42.4)	74 (28.1)
**Detectable HIV viral load, *n*/*N* (%)**	15/17 (88.2)	5/5 (100)	5/6 (83.3)	25/28 (89.3)	57/72 (79.2)
** Missing HIV viral load**	0	0	0	0	2
**Median CD4 cell count, cells/mm^3^ (IQR)**	33.0 (26.0-83.0)	162.0 (147.0-212.0)	156.0 (133.0-160.2)	82.5 (29.8-155.0)	105.0 (48.0-264.0)
** Missing CD4 cell count**	0	0	0	0	1
**Sputum smear grade, *n* (%)**					
** Scanty**	4 (19.0)	4 (13.3)	2 (28.6)	10 (17.2)	28 (14.1)
** 1+**	7 (33.3)	11 (36.7)	2 (28.6)	20 (34.5)	63 (31.8)
** 2+**	1 (4.8)	4 (13.3)	1 (14.3)	6 (10.3)	59 (29.8)
** 3+**	9 (42.9)	11 (36.7)	2 (28.6)	22 (37.9)	48 (24.2)
** Missing sputum smear**	3	3	2	8	65
**Cavitation on chest radiograph, *n* (%)**					
** Yes**	11 (45.8)	19 (57.6)	2 (22.2)	32 (48.5)	113 (43.1)
** No**	12 (50.0)	11 (33.3)	5 (55.6)	28 (42.4)	128 (48.9)
** Uncertain**	1 (4.2)	3 (9.1)	2 (22.2)	6 (9.1)	21 (8.0)
** Missing chest radiograph**	0	0	0	0	1

### Transcriptomic signatures track response to TB treatment

We investigated whether host blood transcriptomic signatures could track treatment response in pulmonary TB by measuring scores at multiple timepoints during treatment. Baseline scores were available for 328 of 329 participants; one sample from a participant with treatment failure was missing. As shown in Figure S1, most signatures were moderately to strongly correlated with each other (Spearman *ρ* ranging from 0.39 to 0.96), suggesting a shared underlying biological signal. Given the strong intercorrelation, we selected Thompson5 and XpertHR as representative examples for figures and analyses because (1) their performance as response biomarkers varied by timepoint, and (2) the Thompson5 signature was the first published parsimonious treatment response signature, while XpertHR has been adapted for a cartridge-based point-of-care assay. Thompson5 and XpertHR were strongly correlated (ρ = 0.65). Notably, Duffy4—a signature of protection from TB in a contained Mtb mouse model^37^—was less correlated with other signatures (*ρ* 0.14-0.57), likely due to its predominant mapping to T-cell gene pathways.

**Figure 2:**
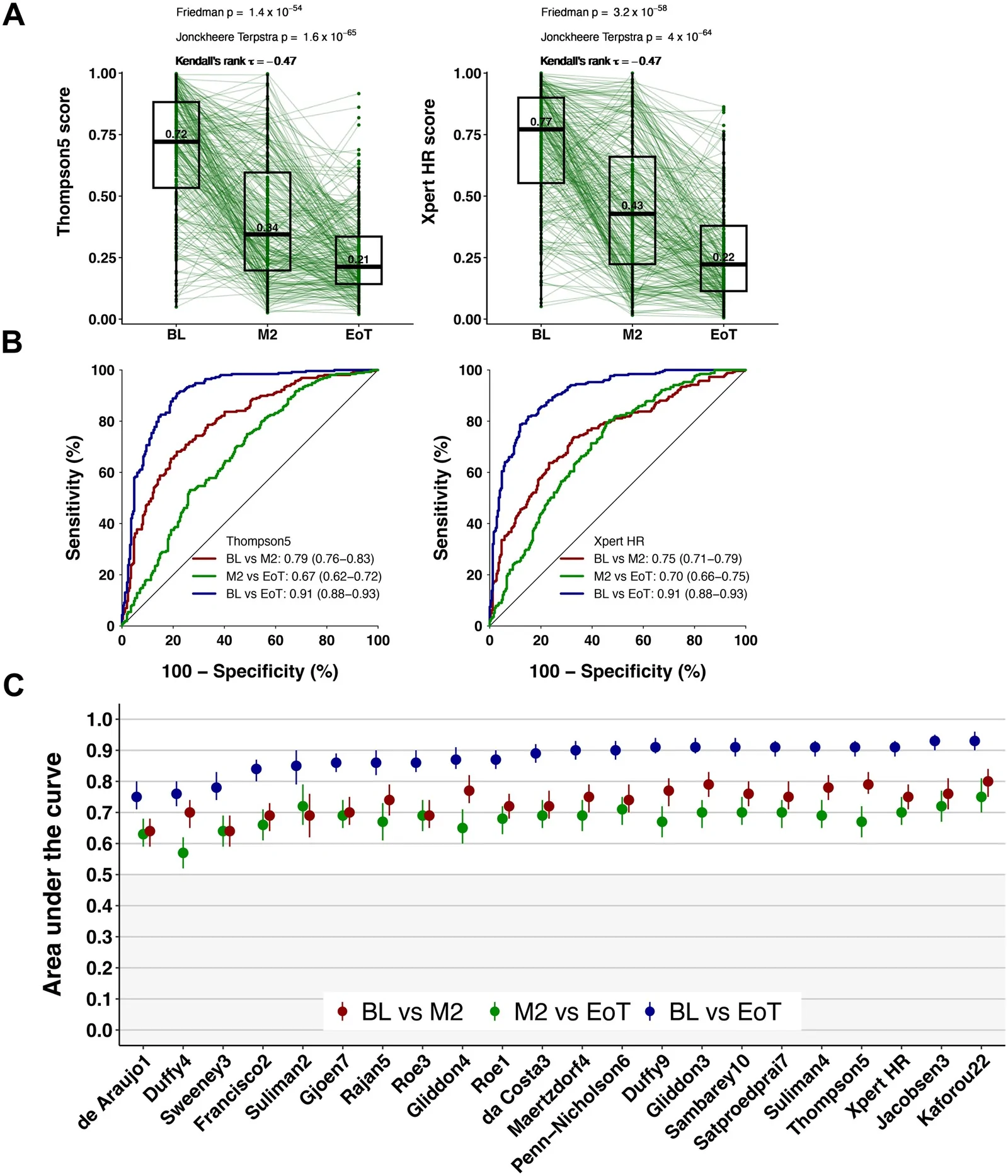
Transcriptomic signatures track response to TB treatment. (A) Line graphs depicting score trajectories of 2 representative signatures, Thompson5 (left) and Xpert Host-Response (HR; right), measured at start (BL = baseline), month 2 (M2), and end of treatment (EoT) for drug-susceptible TB among participants with recurrence-free cure. Each line represents a participant. Boxes depict the IQR, the midline represents the median (shown), and the whiskers indicate the IQR ± (1.5 × IQR). (B) Thompson5 (left) and XpertHR (right) signature receiver operating characteristic (ROC) curves with area under the curve (AUC) estimates and 95% CIs for differentiating paired signature scores measured at the BL versus M2 of treatment (red), M2 versus EoT (green), and BL versus EoT (blue). (C) Summary of ROC performance (AUC) for differentiating paired signature scores measured at the BL versus M2 of treatment (red), M2 versus EoT (green), and BL versus EoT (blue). Signatures are ordered by AUC estimates for BL versus EoT. Circles indicate the AUC estimate and error bars indicate 95% CIs.

We next evaluated signature trajectories over time. Thompson5 and XpertHR (Figure 2A) were highest at baseline and declined progressively during treatment, despite substantial inter-individual variability. These downward trends were confirmed by Friedman and Jonckheere–Terpstra tests (*P* < 1 × 10^−54^) and consistent negative correlations over time (Kendall’s τ = −0.47 for both). Both signatures also showed strong discrimination between treatment stages. They readily distinguished baseline from month 2 samples (AUC ≥ 0.75) and from EoT samples (AUC = 0.91; Figure 2B). To explore whether these patterns extended across the broader set of transcriptomic signatures, we compared AUCs for all 22 signatures across timepoints (Figure 2C). Most signatures showed moderate discrimination between baseline and month 2, weaker separation between month 2 and EoT, and strong discrimination for baseline and EoT (Figure 2C).

### Signatures predicted unfavorable treatment outcomes

**Figure 3:**
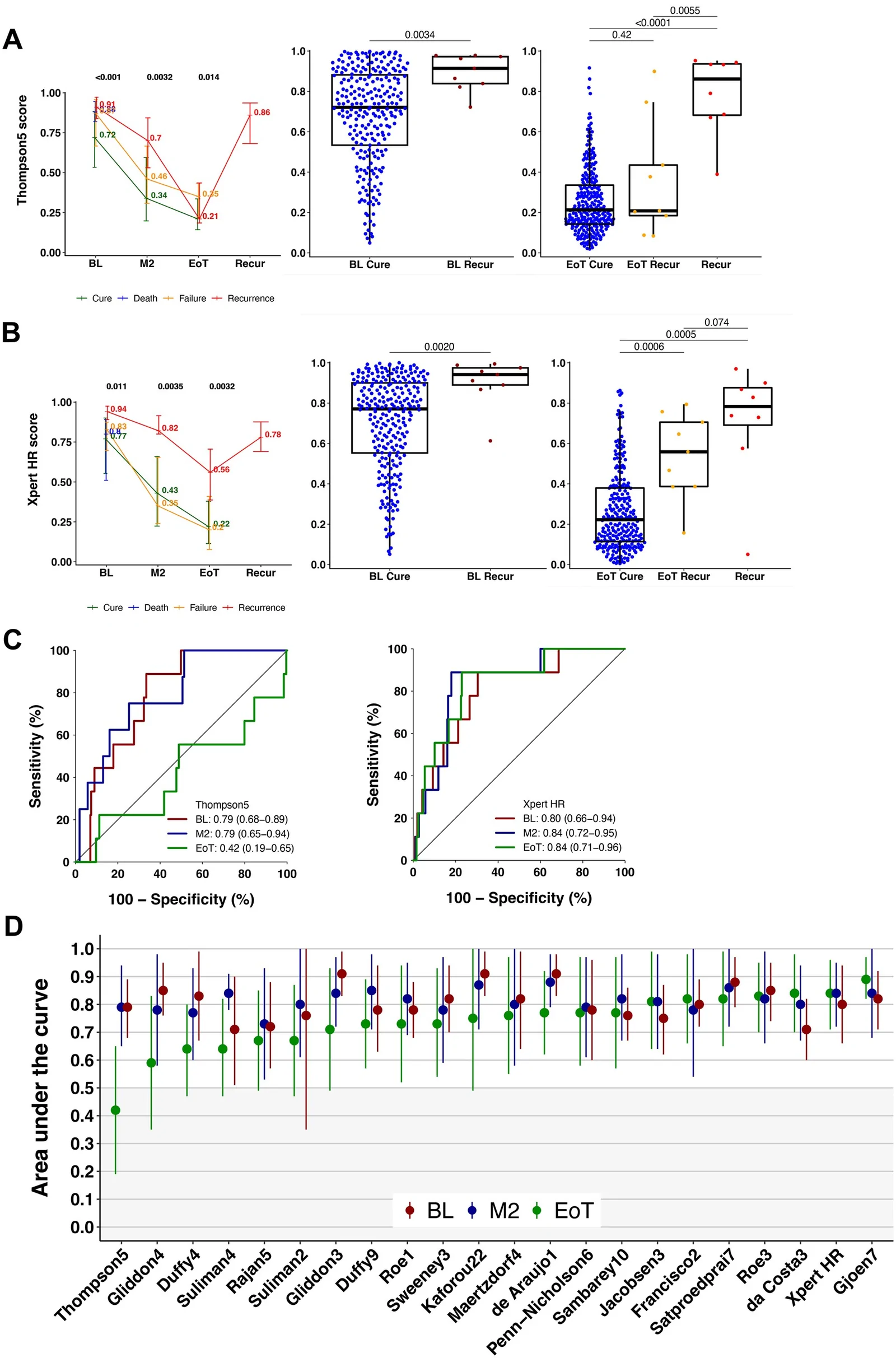
Transcriptomic signatures distinguish individuals with TB recurrence from those with recurrence-free cure. (A, B) Longitudinal changes for 2 representative signatures, Thompson5 (A) and XpertHR (B), among participants with recurrence-free cure (green), recurrence (red), failure (orange), and death (blue). Left panels show median scores (error bars indicate interquartile range) at baseline (BL), month 2 (M2), end of treatment (EoT), and at time of recurrent TB diagnosis (recur). Middle panels compare scores at BL between those with cure (BL cure) vs. recurrence (BL recur). Right panels show EoT scores between those with cure (EoT cure) vs. recurrence (EoT recur), and scores at the time of recurrence (recur). *P* values of between-group differences were computed by Wilcoxon tests. (C) Receiver operating characteristic (ROC) curves evaluating Thompson5 (left) and XpertHR (right) for prediction of recurrence at BL (red), M2 (blue), and EoT (green). Area under the curve (AUC) estimates and 95% CIs are shown. (D) Summary of ROC performance (AUC) for all transcriptomic signatures at BL (red), M2 (blue), and EoT (green), for predicting recurrence. Signatures are ordered by AUC estimates at EoT. Circles indicate the AUC estimate and error bars indicate 95% CIs.

Next, we assessed the performance of transcriptomic signatures to differentiate between individuals who achieved clinical cure and remained disease-free during follow-up and those who experienced unfavorable treatment outcomes. Median signature scores for Thompson5 and XpertHR were lower at all timepoints among individuals with cure, relative to individuals with poor treatment outcomes (Figure 3A and B). Scores for both signatures were significantly higher in individuals who ultimately developed recurrent TB compared to those who achieved cure at baseline and at EoT for XpertHR, but not Thompson5. Consistent with this, XpertHR and Thompson5 predicted recurrence at baseline and month 2, while XpertHR also predicted recurrence at EoT (Figure 3C; Table S4A). Interestingly, relative to EoT, signature scores increased further at the time of recurrent TB diagnosis, especially for the Thompson5 signature (Figure 3A and B). Similar significant prediction of recurrent TB was obtained for virtually all signatures tested, suggesting strongly elevated expression of IFN-response and inflammatory genes among participants at risk of recurrence (Figure 3D; Table S4A).

When benchmarked against the WHO TPP for TB treatment monitoring and optimization (Table S3), 2 of 22 transcriptomic signatures met the minimum prognostic criteria for predicting recurrence at baseline (90% sensitivity, 70% specificity), 13 signatures met the benchmark at month 2 (75% sensitivity, 80% specificity), and none achieved the benchmark at EoT (80% sensitivity, 90% specificity; Table S5). An additional 14 of the 22 signatures had 95% confidence interval upper limits that exceeded the benchmark at baseline, 12 signatures at month 2, and 7 at EoT. The best-performing signature at the EoT for prediction of recurrence was Gjøen7, which achieved a sensitivity of 66.7% (95% CI 35.4-87.9), meaning that approximately two-thirds of recurrences would be detected, with specificity fixed at 90%. Sensitivity fell short of the 80% WHO TPP minimum target. The corresponding PPV was 18.2% (95% CI 8.6-34.4) and NPV 98.7% (95% CI 96.3-99.6), at a recurrence rate of 3.4%. The NNT was 5.5 (95% CI 2.9-11.6), indicating that treatment would need to be prolonged in about 6 patients with elevated scores to potentially prevent one recurrence. At month 2, XpertHR had a sensitivity of 88.9% (95% CI 56.5-98.0) with specificity fixed at 80%, exceeding the WHO TPP, with PPV 13.1% (95% CI 6.8-23.8), NPV 99.5% (95% CI 97.3-99.9), and NNT 7.6 (95% CI 4.2-14.7). At baseline, Gjøen7 had a sensitivity of 88.9% (95% CI 56.5-98.0), approaching the WHO TPP with specificity fixed at 70%, PPV 9.3% (95% CI 4.8-17.3), NPV 99.0% (95% CI 96.6-99.7), and NNT 10.8 (95% CI 5.8-20.9).

**Figure 4:**
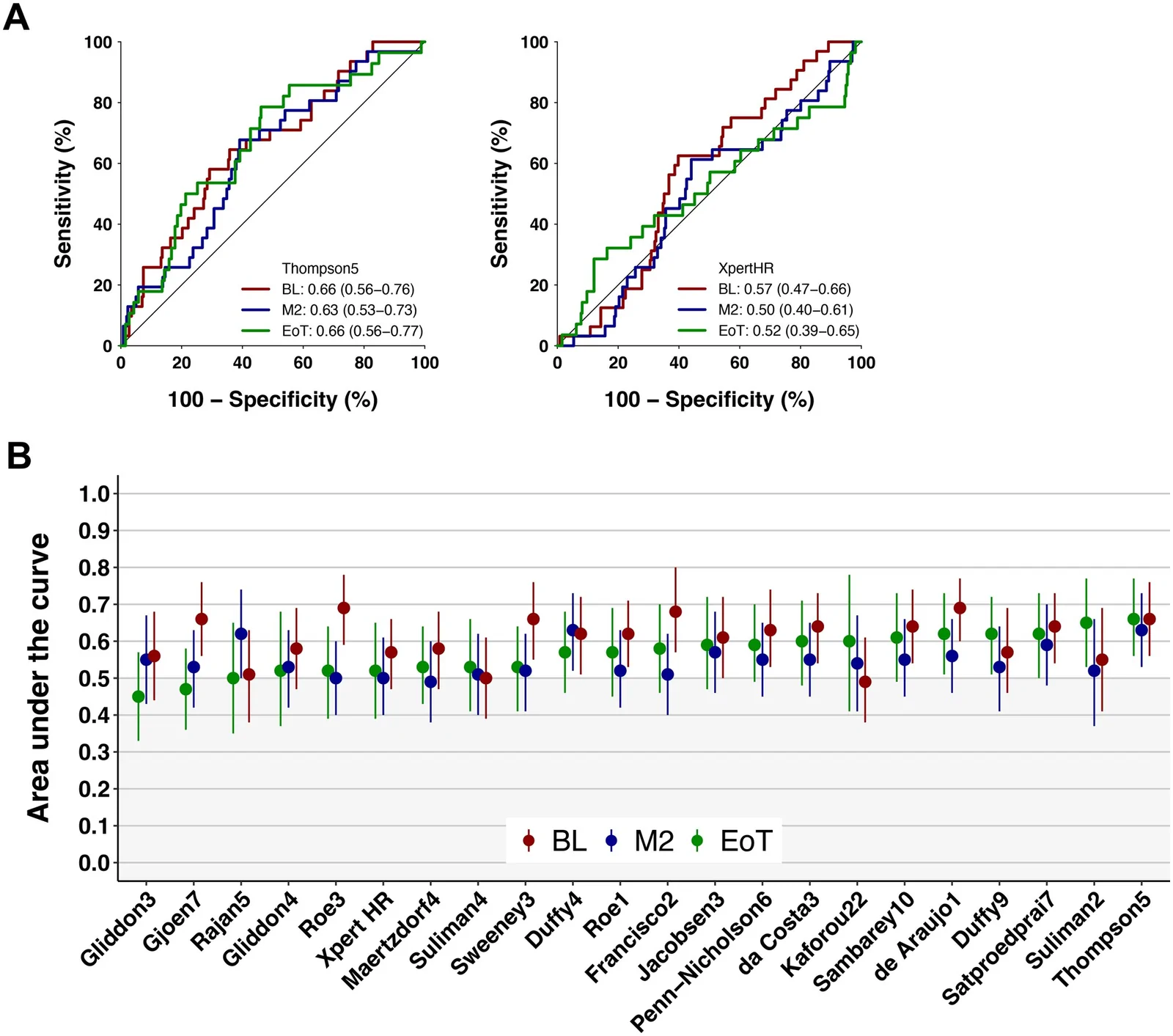
Transcriptomic signature performance for prediction of treatment failure. (A) Receiver operating characteristic (ROC) curves for 2 representative signatures (left: Thompson5; right: XpertHR), comparing individuals who experienced treatment failure versus those with recurrence-free cure, at baseline (BL, red), month 2 (M2, blue), and end of treatment (EoT, green). Area under the curve (AUC) estimates and 95% CIs are shown. (B) Summary ROC performance (AUC) for all transcriptomic signatures at BL (red), M2 (blue), and EoT (green), for predicting treatment failure. Signatures are ordered by AUC estimates at EoT. Circles indicate the AUC estimate and error bars indicate 95% CIs.

We also investigated the performance of transcriptomic signatures for treatment failure. However, the discriminatory performance to predict failure was weak across all timepoints. Thompson5 significantly differentiated between those with treatment failure and cure, but with AUCs not exceeding 0.70 (Figure 4A). The XpertHR signature did not differentiate between those with treatment failure and cure at any timepoint (Figure 4B), a result that was observed for most signatures assessed (Figure 4C; Table S6A). All signatures fell well short of the WHO TPP prognostic criteria at all treatment timepoints (Table S7).

**Figure 5:**
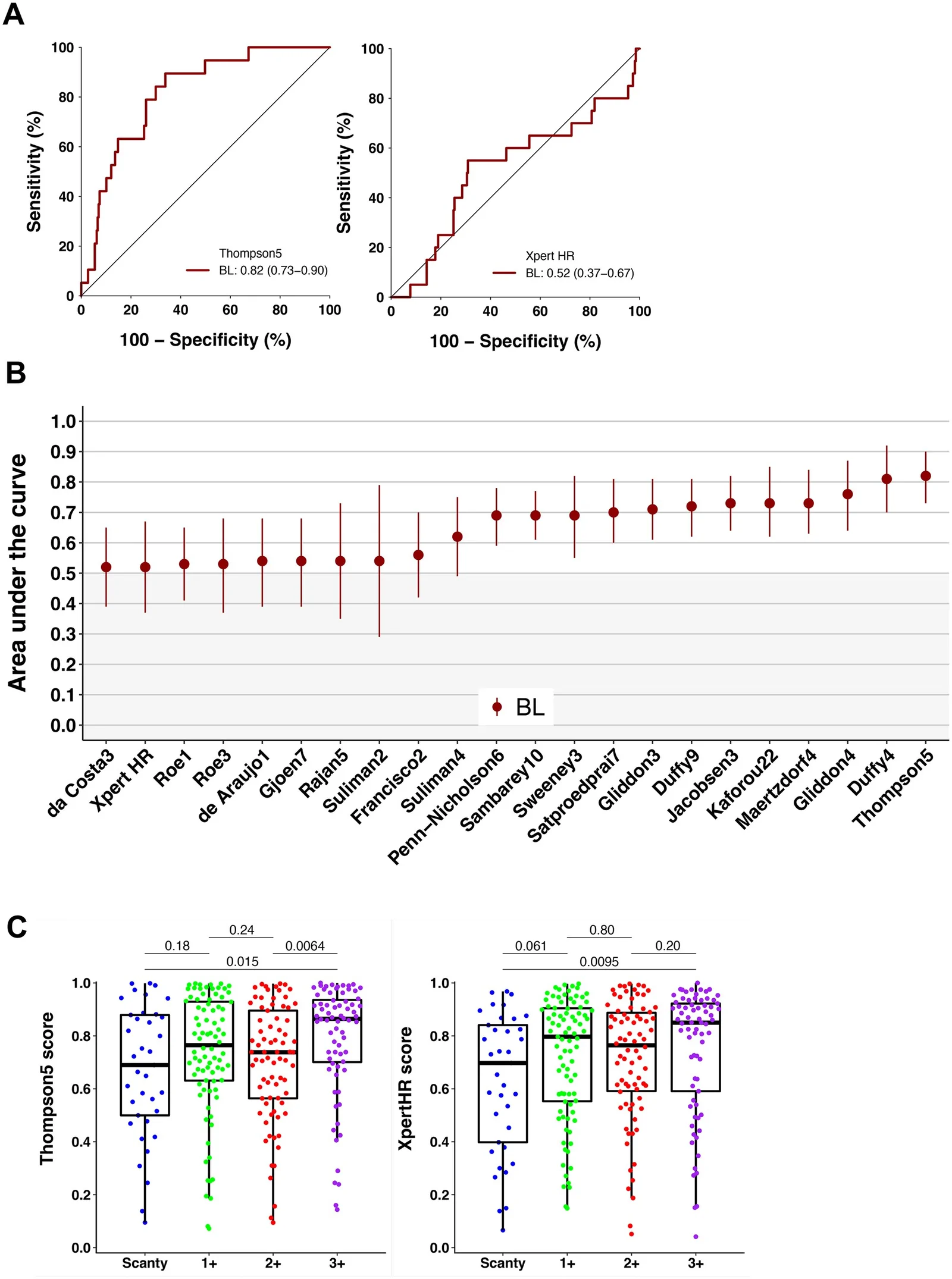
Transcriptomic signatures predict TB-related or unknown-cause death and are associated with sputum bacillary burden. (A) Receiver operating characteristic (ROC) curves for 2 representative signatures (left: Thompson5; right: XpertHR) at baseline (BL) comparing individuals who died during or after treatment to those who achieved recurrence-free cure. Area under the curve (AUC) estimates and 95% CIs are shown. (B) Summary of receiver operating characteristic (ROC) performance (AUC) for all transcriptomic signatures at BL for prediction of TB-related or unknown-cause death. Signatures are ordered by AUC estimates at BL. Circles indicate the AUC estimate and error bars indicate 95% CIs. (C) Boxplots of baseline Thompson5 (left) and XpertHR (right) scores stratified by sputum smear microscopy grade (scanty, 1+, 2+, 3+). P values correspond to pairwise comparisons (Wilcoxon rank-sum tests). Each dot represents a participant. Boxes depict the IQR, the midline represents the median, and the whiskers indicate the IQR ± (1.5 × IQR).

Finally, we determined if transcriptomic signatures could predict death due to TB or unknown cause in this cohort. At baseline, the Thompson5 signature distinguished individuals who later died from those who achieved a recurrence-free cure (Figure 5A, left). In contrast, the XpertHR signature demonstrated no discrimination (Figure 5A, right). When assessed across the full set of signatures at baseline, 11 other signatures also showed significant differentiation between those who ultimately died and controls who remained disease-free (Figure 5B; Table S8A). No signature met the WHO TPP at baseline (90% sensitivity, 70% specificity) for prediction of death (Table S9), but Thompson5 came closest with sensitivity of 84.2% (95% CI 62.4-94.5) at a fixed 70% specificity. To investigate the effect of covariates on signature prognostic performance, we also performed a sensitivity analysis comparing signature-only models to models additionally adjusted for age, sex, and HIV status; covariate adjustment produced endpoint- and timepoint-specific changes in AUC but did not change the overall interpretation of signature performance (Supplementary Results; Tables S4B, S6B, and S8B).

To explore whether elevated transcriptomic scores reflected higher bacterial burden, we stratified Thompson5 and XpertHR scores by sputum smear grade at baseline. Although scores for many signatures, including Thompson5 and XpertHR, were significantly higher in those with smear microscopy scores of 3 + compared to scanty (Figure 5C), the proportions of participants with pulmonary cavities or other commonly used measures of disease severity did not differ. We then performed a multivariable linear regression analysis to identify factors associated with baseline signature scores (Table S10). Independent of treatment outcome, a higher sputum smear grade was associated with elevated baseline scores in 13/22 signatures. No other clinical variables, including HIV status and cavitation, showed consistent associations across signatures.

## Discussion

We evaluated previously published, parsimonious host-blood transcriptomic signatures for their ability to track treatment response and predict recurrent TB, treatment failure, or death among adults with pulmonary TB in Brazil. No validated non-sputum biomarkers currently exist to monitor response or guide regimen choice and duration. To our knowledge, this is the first comprehensive RT-qPCR evaluation of these signatures in a large, well-characterized prospective cohort, reflecting the heterogeneity of real-world treatment response.

Multiple host-blood transcriptomic signatures exhibited dynamic, treatment-responsive trajectories, with scores generally declining over therapy, consistent with resolution of systemic inflammation and immune dysregulation in clinical TB.^18^^,^^38^^,^^39^ Despite substantial inter-individual heterogeneity, the signatures discriminated between samples collected at baseline, month 2, and the EoT. Our findings are consistent with previous studies showing treatment-associated shifts in whole-blood gene expression.^11^^,^^12^^,^^15^^,^^16^^,^^38^^,^^40^ However, whether such signatures can prospectively identify patients at high risk of poor outcomes has remained uncertain.

In our cohort, several parsimonious signatures, most originally developed for TB diagnosis or prognosis, predicted TB recurrence. Additionally, several met the WHO TPP minimum criteria at baseline or month 2, supporting their potential for individualized, biomarker-guided treatment optimization. Such tools could prompt longer treatment for high-risk patients. Conversely, such tools may justify shortening in early responders whose scores normalize toward healthy ranges (consistent with evidence that cure is achievable with shorter regimens^7^^,^^8^), thereby reducing drug exposure and improving adherence and optimizing resource use.

Several signatures also predicted death, indicating that blood-based biomarkers could help identify patients at heightened risk who may benefit from more intensive assessment and care. In our cohort, most deaths occurred among PLHIV (70.8%; median CD4 33 cells/mm^3^, IQR 26-83), disproportionate to their representation among participants with recurrence-free cure (28.1%; median CD4 105 cells/mm^3^, IQR 48-264; Table 2), which may partly explain the strong association between inflammatory activity and mortality. This aligns with findings in HIV-associated TB, where elevated inflammatory proteins predict death.^41^

Notably, the signatures poorly predicted treatment failure across timepoints; this may reflect (1) the predominantly focal nature of bacteriological failure, due to bacilli persisting in poorly perfused lesions with limited systemic inflammatory signal; (2) etiologic heterogeneity (suboptimal adherence, pharmacokinetic variability, drug–drug interactions, or undetected resistance) that is not encoded in host transcriptomic activity; and (3) the relatively small number of failures, reducing statistical power. Collectively, these observations suggest that blood signatures index global inflammatory disease activity—relevant to recurrence and death—better than they capture pharmacologic or microbiologic determinants of early bacteriological failure. However, we note that previous studies have reported transcriptomic signatures that could differentiate between individuals with treatment cure and those with treatment failure.^12^

Transcriptomic scores correlated with bacillary burden, increasing with higher sputum smear grade. Although not uniform across all signatures, this pattern suggests that host-response signatures may partially reflect disease severity. Previous reports have demonstrated that the Sweeney3 and Penn-Nicholson6 signatures correlate with the extent of pulmonary disease quantified by PET-CT imaging.^13^^,^^14^ We observed no associations with cavitation, underscoring that these signals capture systemic inflammatory activity not fully represented by structural radiographic markers.

HIV can shift the baseline transcriptional milieu and the dynamics of TB treatment-associated transcriptional change, so selected transcripts and optimal thresholds may differ by HIV status. Although a single assay that performs across HIV strata is desirable, our limited number of adverse outcomes (particularly recurrence) precluded precise stratified estimates; larger studies should formally test effect modification (including by viral load) and, if present, support HIV-specific calibration or decision thresholds. In sensitivity analyses adjusting for age, sex, and HIV status, discrimination was largely unchanged for recurrence and treatment failure, whereas covariate adjustment more frequently improved AUC for death, consistent with mortality risk being more strongly influenced by demographic and HIV-related factors. This suggests that mortality prediction may benefit from integrated clinical-plus-transcriptomic models.

Our study had limitations. Recurrence events were few, and two-thirds were not microbiologically confirmed, resulting in wide confidence intervals and reducing certainty of endpoint ascertainment, which potentially affected estimates of biomarker performance. The absence of culture confirmation and genotyping also prevented linkage of recurrence isolates to the index strain, precluding classification of recurrence as relapse or reinfection, outcomes with distinct biology that may differentially influence host-response signatures. The absence of Mtb genotyping also precluded assignment of Mtb lineage and exploration of whether host-response signature performance or trajectories vary by lineage. Death due to TB or unknown cause and symptom-triggered (rather than scheduled) post-treatment recurrence assessments may have led to outcome misclassification and under-ascertainment of asymptomatic recurrence. Our analyses were restricted to a risk-score–matched subset, which excluded participants who were lost to follow-up. As a result, diagnostic estimates reflect the matched analytic sample and may not generalize to the full RePORT-Brazil cohort; PPV/NPV and NNT in particular are not prevalence-representative. Despite propensity matching, we did not match on all prognostically important characteristics (eg, cavitation, smear grade), and residual confounding by measured and unmeasured factors may persist. To mitigate overfitting, we used a previously optimized RT-qPCR protocol, calculated signature scores with locked-down models without retraining on the present cohort and remained blinded to clinical phenotypes throughout. Nevertheless, we evaluated 22 signatures and highlighted those performing best in this dataset; generalizability to other settings and populations remains to be established.

In summary, parsimonious host-blood transcriptomic signatures show promise as biomarkers to monitor treatment response and to prognosticate poor outcomes in pulmonary TB. A necessary next step is to validate that such signatures predict relapse or failure in the setting of treatment-shortening regimens (eg, the 4-month rifapentine–moxifloxacin regimen from TBTC Study 31/ACTG A5349),^7^ using retrospective analyses or prospectively embedded studies. Contingent on positive validation, a pragmatic biomarker-guided trial, assigning longer or shorter regimens on the basis of pre-specified high/low signature thresholds would then be warranted to establish clinical utility and safety.^42^ An optimal test would be non-sputum (ideally peripheral/finger-prick blood), accurately reflect disease activity and bacillary clearance across therapy, and be especially valuable where sputum is hard to obtain. Used alongside standard clinical and microbiological assessment, such signatures could facilitate precision care by supporting regimen-shortening for early responders and targeted intensification for high-risk patients (eg, additional drugs or extended treatment duration). Our results provide rationale and candidate signatures for advancing these biomarkers into pragmatic trials of individualized TB treatment.

## Supplementary Material

aamag156_Supplementary_Data

## Data Availability

Deidentified qPCR data, signature scores, and TB endpoint data underlying the results have been deposited in Zivahub (doi.org/10.25375/uct.30121768), an open access data repository hosted by the University of Cape Town’s institutional data repository powered by Figshare for Institutions. Additional clinical metadata are available on request from the RePORT Consortium (reportinternational.org).
