# Supplementary material for: Blood transcriptomic signatures predict poor outcomes in drug-susceptible pulmonary tuberculosis in Brazil
Source: Am J Respir Crit Care Med. 2026 Mar 30;212(9):2211–25. doi: 10.1093/ajrccm/aamag156 (PMC13519344; doi:10.1093/ajrccm/aamag156)
Supplement: Supplementary file 1 [file aamag156_supplementary_data.zip › coi_disclosure_VR.pdf]

ICMJE DISCLOSURE FORM

**Date:**	01/30/2026
**Your Name:**	Valeria Cavalcanti Rolla
**Manuscript Title:**	Blood transcriptomic signatures predict poor outcomes in drug-susceptible pulmonary TB in Brazil
**Manuscript Number (if known):**	Blue-202510-2389OC

In the interest of transparency, we ask you to disclose all relationships/activities/interests listed below that are related to the content of your manuscript. "Related" means any relation with for-profit or not-for-profit third parties whose interests may be affected by the content of the manuscript. Disclosure represents a commitment to transparency and does not necessarily indicate a bias. If you are in doubt about whether to list a relationship/activity/interest, it is preferable that you do so.

The author's relationships/activities/interests should be defined broadly. For example, if your manuscript pertains to the epidemiology of hypertension, you should declare all relationships with manufacturers of antihypertensive medication, even if that medication is not mentioned in the manuscript.

In item #1 below, report all support for the work reported in this manuscript without time limit. For all other items, the time frame for disclosure is the past 36 months.

		Name all entities with whom you have this relationship or indicate none (add rows as needed)	Specifications/Comments (e.g., if payments were made to you or to your institution)
		**Time frame: Since the initial planning of the work**	
1	All support for the present manuscript (e.g., funding, provision of study materials, medical writing, article processing charges, etc.)<br>**No time limit for this item.**	☐ None	
		Departamento de Ciência e Tecnologia (DECIT) - Secretaria de Ciência e Tecnologia (SCTIE) – Ministério da Saúde (MS), Brazil	25029.000507/2013-07 to Fiotec
		National Institutes of Health/National Institutes of Allergy and Infectious Diseases (NIH/NIAID)	U01 AI069923; R01 A1120790; F31 AI152614 to L.S.P
			Click the tab key to add additional rows.
		**Time frame: past 36 months**	
2	Grants or contracts from any entity (if not indicated in item #1 above).	☐ None	
		TB Alliance for Drug Development	NC-008
		Impacto da COVID-19 Nas Manifestações Clínicas, Diagnóstico, Desfecho Do Tratamento e Resposta Imune para Tuberculose Pulmonar	440933/2020-0 to CNPq account (not me)
		Associative BRICS Research in COVID-19 and Tuberculosis (ABRICOT)	G-202105-67821 To Fiotec
		RePORT International Data Harmonization	R-202108-68053 to Fiotec
		Transcriptional Signature of TB in advanced HIV	DAA3-18-64313 to Fiotec
		Predictors of Resistance Emergence Evaluation in Multidrug-resistant Tuberculosis patients on Treatment - PREEMPT	R01AI134430

		Name all entities with whom you have this relationship or indicate none (add rows as needed)	Specifications/Comments (e.g., if payments were made to you or to your institution)
		Caribbean, Central, and South America network for HIV Epidemiology (CCASAnet)	U01AI069923 to fiotec
		Immunogenetic predictors of active and incipient TB in HIV-negative and -positive close TB contacts	R01AI147765 to fiotec
3	Royalties or licenses	☒ **None**	
4	Consulting fees	☐ **None**	
		ONU- OMS Country review for HIV response Mianmar	Payment made to me
5	Payment or honoraria for lectures, presentations, speakers bureaus, manuscript writing or educational events	☐ **None**	
		**GLAXOSMITHKLINE BRASIL LTDA**	Payment made to me
		Qiagen (Infecto Rio Conference)	Payment made to me
		Virology education (Infecto Rio Conference)	Payment made to me
6	Payment for expert testimony	☒ **None**	
7	Support for attending meetings and/or travel	☐ **None**	
		ViiV 18 European AIDS conference	Registration - payment direct to the conference
8	Patents planned, issued or pending	☒ **None**	

		Name all entities with whom you have this relationship or indicate none (add rows as needed)	Specifications/Comments (e.g., if payments were made to you or to your institution)
**9**	Participation on a Data Safety Monitoring Board or Advisory Board	☐ **None**	
		NIH Data Safety Monitoring board	Payment direct to me
**10**	Leadership or fiduciary role in other board, society, committee or advocacy group, paid or unpaid	☒ **None**	
**11**	Stock or stock options	☒ **None**	
**12**	Receipt of equipment, materials, drugs, medical writing, gifts or other services	☒ **None**	
**13**	Other financial or non-financial interests	☒ **None**	

Please place an "X" next to the following statement to indicate your agreement:

☒ I certify that I have answered every question and have not altered the wording of any of the questions on this form.
