# Supplementary material for: Blood transcriptomic signatures predict poor outcomes in drug-susceptible pulmonary tuberculosis in Brazil
Source: Am J Respir Crit Care Med. 2026 Mar 30;212(9):2211–25. doi: 10.1093/ajrccm/aamag156 (PMC13519344; doi:10.1093/ajrccm/aamag156)
Supplement: Supplementary file 1 [file aamag156_supplementary_data.zip › TranscriptomicSigs_Supplementary_20260130_CLEAN.docx]

**Supplementary Material**

[RePORT South Africa and RePORT-Brazil Consortia	3](#_Toc221793745)

[Supplemental Methods	4](#_Toc221793746)

[Propensity (or risk) scores	4](#_Toc221793747)

[Transcriptomic signature panel design and measurement	4](#_Toc221793748)

[Data quality control	4](#_Toc221793749)

[Signature score calculation	5](#_Toc221793750)

[Signature reparameterisation	5](#_Toc221793751)

[Batch correction and score normalisation	6](#_Toc221793752)

[Blinding	6](#_Toc221793753)

[Sensitivity analyses adjusting for age, sex, and HIV status	6](#_Toc221793754)

[Supplemental Results	7](#_Toc221793755)

[Sensitivity analyses adjusting for age, sex, and HIV status	7](#_Toc221793756)

[Supplemental Figures	8](#_Toc221793757)

[Figure S1. Correlation between signature scores.	8](#_Toc221793758)

[Supplemental Tables	9](#_Toc221793759)

[Table S1. TaqMan PCR primer-probe panel for the 96.96 gene expression integrated fluidic circuit.	9](#_Toc221793760)

[Table S2. Transcriptomic signatures on the panel that were reparameterised.	11](#_Toc221793761)

[Table S3. Target Product Profile for TB treatment biomarkers.	12](#_Toc221793762)

[Table S4A. Performance (area under the curve) of transcriptomic signatures measured at baseline, month 2, and end of treatment (month 6) for predicting TB recurrence after completion of treatment.	13](#_Toc221793763)

[Table S4B. Sensitivity analysis: Performance of transcriptomic signatures measured at baseline, month 2, and end of treatment (month 6), versus covariate-adjusted signature models, for predicting TB recurrence after completion of treatment.	14](#_Toc221793764)

[Table S5. Sensitivity, specificity, positive predictive value, and negative predictive value of transcriptomic signatures measured at baseline, month 2, and end of treatment (month 6) for predicting TB recurrence after completion of treatment.	15](#_Toc221793765)

[Table S6A. Performance (area under the curve) of transcriptomic signatures measured at baseline, month 2, and end of treatment (month 6) for predicting TB treatment failure.	16](#_Toc221793766)

[Table S6B. Sensitivity analysis: Performance of transcriptomic signatures measured at baseline, month 2, and end of treatment (month 6), versus covariate-adjusted signature models, for predicting TB treatment failure.	17](#_Toc221793767)

[Table S7. Sensitivity, specificity, positive predictive value, and negative predictive value of transcriptomic signatures measured at baseline, month 2, and end of treatment (month 6) for predicting TB treatment failure.	18](#_Toc221793768)

[Table S8A. Performance (area under the curve) of transcriptomic signatures measured at baseline for predicting death due to TB or unknown cause.	19](#_Toc221793769)

[Table S8B. Sensitivity analysis: Performance of transcriptomic signatures measured at baseline, versus covariate-adjusted signature models, for predicting death due to TB or unknown cause.	20](#_Toc221793770)

[Table S9. Sensitivity, specificity, positive predictive value, and negative predictive value of transcriptomic signatures measured at baseline for predicting death due to TB or unknown cause.	21](#_Toc221793771)

[Table S10. Multivariable linear regression analysis to identify factors associated with baseline Thompson5 and XpertHR signature scores.	22](#_Toc221793772)

[Supplemental References	23](#_Toc221793773)

# RePORT South Africa and RePORT-Brazil Consortia

*RePORT-South Africa Consortium*

Nicole Bilek

Yolundi Cloete

Katie Hadley

Rieyaat Hassiem

Lungisa Jaxa

Stanley Kimbung Mbandi

Faheemah Meyer

Humphrey Mulenga

Onke Nombida

Alison September

Ashley Veldsman

*RePORT-Brazil Consortium*

Brenda Carvalho

Adriano Gomes

Cody Staats

Megan Turner

# Supplemental Methods

## Propensity (or risk) scores

Propensity (or risk) scores^1^ were estimated using logistic regression. Age was included as a continuous covariate in the propensity/risk score model, while sex, HIV status, and site of enrolment were used as exact matching variables. Matching was performed using nearest-neighbour matching on the estimated propensity/risk score, with a caliper of 0.25—that is, participants with unfavourable outcomes were only matched to those with favourable outcomes whose propensity scores were within 0.25 standard deviations. Two matching procedures were conducted. First, participants with recurrence-free cure were matched, without replacement, at a 3:1 ratio to each subgroup with unfavourable outcomes (treatment failure, recurrence, and death) to enable outcome-specific contrasts. Second, a 3:1 matching was performed between participants with favourable outcomes and those with any combined unfavourable outcome to increase power and yield a single overall estimate comparable to composite endpoints used in TB studies. All matched participants from both procedures were included in the analysis.

## Transcriptomic signature panel design and measurement

RNA plates were thawed in batches, cDNA synthesised with EpiScript reverse transcriptase (Lucigen, USA), and genes of interest pre-amplified using pools of TaqMan primer-probe assays (Thermo Fisher Scientific, USA; **Table S1**). Gene expression (raw cycle threshold, Ct) was quantified by microfluidic multiplex real-time quantitative PCR (RT-qPCR) using Standard BioTools (formerly Fluidigm, USA) 96.96 (96 samples multiplexed with 96 primer-probe assays) Gene Expression chips on a BioMark HD instrument (Standard BioTools). Quality control filters, batch correction, and calculation of the signature scores were performed using a pre-defined R script.

## Data quality control

Gene expression data were analysed using a pre-defined R script with quality control filters that assessed the integrity and reproducibility of each chip. The following parameters were applied for extracting Ct values: Linear (Derivative) baseline correction, Quality Threshold of 0.3, and Auto (Global) for Ct Threshold Method using Biomark software version 4.5.2. No-template (water) and internal positive control samples were run on each chip. Chips with marked deviation (>5 standard deviations, Spearman correlation < 0.98, or concordance correlation coefficient < 0.95) in the internal positive control sample primer-probe assay raw Ct values or Penn-Nicholson6 (RISK6) signature score versus 11 historical runs, with amplification (raw Ct < 35 cycles) for any assay in the no-template control, or with more than 10% failed primer-probes, were repeated. Individual samples with more than 20% failed primer-probe reactions were classified as failed and no signature scores were computed. If less than 20% of primer-probe reactions failed for an individual sample, signature scores were computed where possible and signatures with missing primer-probe raw Ct values were deemed failed for that sample. Samples and primer-probe assays were run in singlet, and failed signature results for individual samples were assumed to follow a random distribution, thus not repeated, and excluded from analysis.

## Signature score calculation

Signature scores were calculated from raw Ct measurements using the original algorithms developed for each individual signature, where possible (**Table 1**). Signatures discovered using methods other than RT-qPCR, such as RNA-sequencing or microarray, were subsequently re-parameterised to RT-qPCR.^2,3^ In cases where predesigned TaqMan primer-probe assays were not available from the original publication, we used either inventoried primer-probes or designed novel TaqMan assays based on the published Illumina probe, RNA-seq transcript, or primer-probe sequence data. Furthermore, we contacted the authors of relevant publications when the required information was not available. We included three reference primer-probes (ACTR3, TMBIM6, and USF2; **Table S1**) for standardisation of gene expression for signatures which require a normalised (delta) Ct.

## Signature reparameterisation

Eight signatures (da Costa3, Duffy9, Francisco2, Gjøen7, Jacobsen3, Roe3, Sambarey10, and Satproedprai7) developed using gene expression analysis techniques other than RT-qPCR required reparameterisation in order to compute signature scores from microfluidic multiplex RT-qPCR BioMark HD readouts. This was necessary because different platforms each have unique dynamic ranges and quantitative scales for quantifying gene expression levels. The machine learning models, and statistical methods used for computing signature scores therefore needed to be reconstructed (reparameterised) using Ct values generated from RT-qPCR data on the BioMark HD platform. This ensured that there was uniformity in the way scores were calculated for different signatures and how results were interpreted. The model parameters for the eight signatures were reconstructed using the original published methods^4-11^ to best differentiate between TB cases and controls when adapted to Ct values, generated using PAXgene RNA samples from a previously described cross-sectional TB cohort (CTBC)^12,13^. This cohort is completely independent of the RePORT-Brazil study and included both HIV positive and HIV negative TB cases and healthy controls; use of this independent dataset for reparameterisation reduced potential bias. The RT-qPCR protocol for the reparameterisation experiments was consistent with the methods used for all RT-qPCR in this paper.

Model training was done on individuals who were HIV negative (59 LTBI; 50 TB). 70% of HIV negative individuals (42 LTBI; 34 TB) made up the training set and the remaining 30% comprised the test set (17 LTBI; 16 TB). Once a model constructed on the training set demonstrated discrimination on the test set, then both sets were combined to reparameterise on the entire dataset (**Table S2**). Receiver operating characteristic (ROC) curves were generated to validate the performance of each model’s ability to separate HIV-positive TB cases (n=39) from HIV-positive LTBI controls (n=36) (**Table S2**).

A number of signatures had not previously been evaluated using the Biomark RT-qPCR instrument, but did not require reparameterisation even though they were discovered using approaches other than RT-qPCR. Examples include the Gliddon3/4,^14^ Kaforou25,^15^ and Rajan5^16^ signatures. Gliddon3/4 and Kaforou25 were discovered using microarray data using a simple Disease Risk Score (DRS), which sums normalized expression values from all upregulated transcripts and subtracts the normalized expression values of all downregulated transcripts, making the signature essentially “self-normalizing”. For Gliddon4, the original publication reported GBP6 and PRDM1 as upregulated and TMCC1 and ARG1 as downregulated in TB; however, when measured using the same TaqMan assays on our microfluidic RT-qPCR platform, ARG1 consistently showed higher expression in TB (lower Ct) while PRDM1 was not consistently differentially expressed across cohorts. Therefore, consistent with the DRS framework, we calculated the Gliddon4 score as (_deltaCt_TMCC1 + _deltaCt_PRDM1) − (_deltaCt_GBP6 + _deltaCt_ARG1). Rajan5 was also discovered based on microarray gene expression data, but developed using the signed and unsigned sums methods, which also allowed the signature to be “self-normalising”.

## Batch correction and score normalisation

Batch correction was performed to correct for differences in signature score distribution between microfluidic chips using a quantile regression method with the adjust_batch function in the batchtma R package.^17^ This method unifies lower quantiles (25th percentile) and ranges between the lower and upper quantile (75th percentile) between batches, allowing for differences in both parameters due to confounders. Thereafter, signature scores were winsorized (clipped) to account for extreme outliers (Winsorize function, DescTools R package^18^), log transformed to normalise score distribution (log function, base R package), converted to z-scores (scale function, base R package), and compared using the cumulative distribution function (pnorm function, stats R package). De Araujo1, Rajan5, and Roe1 signatures were multiplied by −1 to obtain a positive correlation for all signatures.

## Blinding

Participants and study staff responsible for TB investigation in Brazil were blinded to transcriptomic signature scores. Transcriptomic signature scores were measured by laboratory personnel in South Africa who were blinded to participant treatment outcome. Signature scores and TB microbiology results were maintained in different files, which were integrated only after the study database had been cleaned and locked and the RT-qPCR data was cleaned and finalised.

## Sensitivity analyses adjusting for age, sex, and HIV status

As a sensitivity analysis, we compared signature-only models to models including signature + age + sex + HIV status for each endpoint and timepoint. AUCs were calculated from model-predicted probabilities and compared using DeLong’s test for correlated ROC curves.

# Supplemental Results

## Sensitivity analyses adjusting for age, sex, and HIV status

AUC estimates based on transcriptomic signature scores alone do not indicate how signatures perform relative to, or in combination with, routinely available clinical predictors. To address this, we performed a sensitivity analysis in which we compared **signature-only models** with models including **signature + age + sex + HIV status**. Because controls were selected using propensity-score methods and subsequently pooled for analysis, covariate effects could still influence discrimination; therefore, this covariate-adjusted analysis provides a direct assessment of whether inclusion of key demographic/HIV covariates materially changes the ROC-based comparisons.

For each endpoint (recurrence, failure, and death) and timepoint (baseline, month 2, and end of treatment), we calculated AUCs from model-predicted probabilities and compared AUCs using DeLong’s test for correlated ROC curves. The incremental change in signature discrimination after adjustment for covariates (ΔAUC) was endpoint- and timepoint-specific:

- **Recurrence (Table S4B):** ΔAUC ranged from 0.01 to 0.16 at baseline (3/22 signatures showing significant AUC changes with p<0.05 and 95%CI not crossing zero), −0.04 to 0.11 at month 2 (0/22 showing significant AUC changes), and −0.01 to 0.35 at end of treatment (1/22 showing significant AUC changes).
- **Treatment failure (Table S6B):** ΔAUC ranged from −0.01 to 0.16 at baseline (0/22 showing significant AUC changes), 0.03 to 0.15 at month 2 (5/22 showing significant AUC changes), and 0.03 to 0.25 at end of treatment (1/22 showing significant AUC changes).
- **Death due to TB or unknown causes (Table S8B):** ΔAUC ranged from 0.03 to 0.21 at baseline, with 13/22 signatures showing significant AUC gains.

Adjustment produced minimal changes in discrimination for recurrence and treatment failure across timepoints (with few significant ΔAUC gains), whereas for death, AUC gains with adjustment were more frequent. This pattern is consistent with mortality in this cohort being more strongly influenced by demographic and HIV-related risk, such that integrated clinical-plus-signature models may be preferable for death prediction. These findings support that our main comparative conclusions for recurrence and failure are not explained by omission of HIV status from ROC models, while emphasizing that adequately powered datasets are needed to test effect modification and determine whether HIV-specific thresholds or signatures are warranted.

# Supplemental Figures

## Figure S1. Correlation between signature scores.

Signature score correlation matrix with the Spearman-rank-order correlation coefficients (*rho*) among samples from TB patients, at all timepoints (n= 403 ^#^). De Araujo1, Rajan5, and Roe1 signatures were multiplied by −1 to obtain a positive correlation for all signatures.

^#^Only participants with scores available for all signatures were included in the Spearman correlation analysis.

Xpert HR=Xpert Host-Response.

# Supplemental Tables

## Table S1. TaqMan PCR primer-probe panel for the 96.96 gene expression integrated fluidic circuit.

| **Gene Symbol** | **TaqMan Assay ID** | **Assay excluded**** | **Reference probe** | **# of signatures** | **da Costa3** **^4^** | **de Araujo1** **^19^** | **Duffy4 (5)#** **^20^** | **Duffy9 (10)†^5^** | **Francisco2** **^6^** | **Gjøen7** **^7^** | **Gliddon3** **^14^** | **Gliddon4 ^14^** | **Jacobsen3 ^8^** | **Kaforou22 (27)‡** **^15^** | **Maertzdorf4 ^21^** | **Penn-Nicholson6 ^13^** | **Rajan5 ^16^** | **Roe1 ^22^** | **Roe3 ^9^** | **Sambarey10 ^10^** | **Satproedprai7 ^11^** | **Suliman2 ^23^** | **Suliman4 ^23^** | **Sweeney3 ^24,25^** | **Thompson5 ^26^** | **Xpert HR ^27^** |
| --- | --- | --- | --- | --- | --- | --- | --- | --- | --- | --- | --- | --- | --- | --- | --- | --- | --- | --- | --- | --- | --- | --- | --- | --- | --- | --- |
| ACTR3 | Hs01029159_g1 |  | X | 15 | X | X | X | X | X | X | X | X | X | X |  |  | X | X | X | X | X |  |  |  |  | X |
| TMBIM6 | Hs00162661_m1 |  | X | 15 | X | X | X | X | X | X | X | X | X | X |  |  | X | X | X | X | X |  |  |  |  | X |
| USF2 | Hs01100994_g1 |  | X | 15 | X | X | X | X | X | X | X | X | X | X |  |  | X | X | X | X | X |  |  |  |  | X |
| ACTA2 | Hs00426835_g1 |  |  | 1 |  |  |  |  |  |  |  |  |  |  |  |  | X |  |  |  |  |  |  |  |  |  |
| ANKRD22 | Hs00944015_m1 |  |  | 2 |  |  |  |  |  |  |  |  |  | X |  |  |  |  |  |  |  | X |  |  |  |  |
| APOL1 | Hs01066280_m1 |  |  | 1 |  |  |  |  |  |  |  |  |  |  |  |  |  |  |  |  | X |  |  |  |  |  |
| ARG1 | Hs00968979_m1 |  |  | 1 |  |  |  |  |  |  |  | X |  |  |  |  |  |  |  |  |  |  |  |  |  |  |
| BATF2 | Hs00912737_m1 |  |  | 2 |  |  |  |  |  |  |  |  |  |  |  |  |  | X | X |  |  |  |  |  |  |  |
| BCL6 | AREPURD* |  |  | 1 |  |  |  |  |  |  |  |  |  |  |  |  |  |  |  | X |  |  |  |  |  |  |
| BLK | Hs01017452_m1 |  |  | 1 |  |  |  |  |  |  |  |  |  |  |  |  |  |  |  |  |  |  | X |  |  |  |
| C1QA | Hs00706358_s1 | X |  | 0 |  |  |  |  |  |  |  |  |  |  |  |  |  |  |  |  |  |  |  |  |  |  |
| C1QB | Hs00608019_m1 |  |  | 2 |  |  |  | X |  |  | X |  |  | ‡ |  |  |  |  |  |  |  |  |  |  |  |  |
| C4ORF18 / FAM198B | Hs00259260_s1 |  |  | 1 |  |  |  |  |  |  |  |  |  | X |  |  |  |  |  |  |  |  |  |  |  |  |
| C5 | Hs00156197_m1 |  |  | 1 |  |  |  |  |  |  |  |  |  | X |  |  |  |  |  |  |  |  |  |  |  |  |
| CCR5 | Hs99999149_s1 |  |  | 1 |  |  | X |  |  |  |  |  |  |  |  |  |  |  |  |  |  |  |  |  |  |  |
| CCR6 | Hs01890706_s1 |  |  | 1 |  |  |  |  |  |  |  |  |  | X |  |  |  |  |  |  |  |  |  |  |  |  |
| CD160 | Hs00199894_m1 |  |  | 1 |  |  |  | X |  |  |  |  |  |  |  |  |  |  |  |  |  |  |  |  |  |  |
| CD1C | Hs00957534_g1 |  |  | 1 |  |  |  |  |  |  |  |  |  |  |  |  |  |  |  |  |  |  | X |  |  |  |
| CD36.2 | Hs01567186_m1 |  |  | 1 |  |  |  | X |  |  |  |  |  |  |  |  |  |  |  |  |  |  |  |  |  |  |
| CD3E | APDJ3NT* |  |  | 1 |  |  |  |  |  | X |  |  |  |  |  |  |  |  |  |  |  |  |  |  |  |  |
| CD40L | Hs00163934_m1 |  |  | 1 |  |  |  | X |  |  |  |  |  |  |  |  |  |  |  |  |  |  |  |  |  |  |
| CD74-j1 | Hs04983808_s1 | X |  | 0 |  |  |  |  |  |  |  |  |  |  |  |  |  |  |  |  |  |  |  |  |  |  |
| CD74-j2 | AP9HN47* | X |  | 0 |  |  |  |  |  |  |  |  |  |  |  |  |  |  |  |  |  |  |  |  |  |  |
| CD79A | Hs00998120_g1 |  |  | 1 |  |  |  |  |  |  |  |  |  | X |  |  |  |  |  |  |  |  |  |  |  |  |
| CD79B | Hs01058826_g1 |  |  | 1 |  |  |  |  |  |  |  |  |  | X |  |  |  |  |  |  |  |  |  |  |  |  |
| CDKN1C | Hs00175938_m1 | X |  | 0 |  |  |  |  |  |  |  |  |  |  |  |  |  |  |  |  |  |  |  |  |  |  |
| CXCR5 | Hs00540548_s1 |  |  | 1 |  |  |  |  |  |  |  |  |  | X |  |  |  |  |  |  |  |  |  |  |  |  |
| CYP4F3 | Hs01587860_mH |  |  | 1 |  |  |  |  |  |  |  |  |  |  |  |  |  |  |  | X |  |  |  |  |  |  |
| DUSP3 | Hs01115776_m1 |  |  | 2 |  |  |  |  |  |  |  |  |  | X |  |  |  |  |  |  |  |  |  | X |  | X |
| FAM20A | Hs01034066_m1 |  |  | 1 |  |  |  |  |  |  |  |  |  | X |  |  |  |  |  |  |  |  |  |  |  |  |
| FCGR1A | Hs00174081_m1 |  |  | 5 | X |  |  |  |  |  | X |  | X | X |  |  |  |  |  | X |  |  |  |  |  |  |
| FCGR1A | AP2XDMK* |  |  | 1 |  |  |  |  |  |  |  |  |  |  |  |  |  |  |  |  | X |  |  |  |  |  |
| FCGR1B | Hs02341825_m1 |  |  | 3 |  |  |  | X |  |  |  |  |  | X |  | X |  |  |  |  |  |  |  |  |  |  |
| FCGR1B_ VARIANT1 | AP3267H* |  |  | 1 |  |  |  |  |  |  |  |  |  |  |  |  |  |  |  |  | X |  |  |  |  |  |
| FCGR1B_ VARIANT2 | AP47ZTF* |  |  | 1 |  |  |  |  |  |  |  |  |  |  |  |  |  |  |  |  | X |  |  |  |  |  |
| FCGR1C | Hs00417598_m1 |  |  | 1 |  |  |  |  |  |  |  |  |  | X |  |  |  |  |  |  |  |  |  |  |  |  |
| FLVCR2 | Hs00900390_m1 |  |  | 1 |  |  |  |  |  |  |  |  |  | X |  |  |  |  |  |  |  |  |  |  |  |  |
| GAS6 | AR47XGZ* |  |  | 2 |  |  |  |  |  |  |  |  |  | X |  |  |  |  |  |  |  |  | X |  |  |  |
| GAS6 | AR323W3* |  |  | 1 |  |  |  |  |  |  |  |  |  | X |  |  |  |  |  |  |  |  |  |  |  |  |
| GBP1 | Hs00977005_m1 |  |  | 1 |  |  |  |  |  |  |  |  |  |  | X |  |  |  |  |  |  |  |  |  |  |  |
| GBP2 | Hs00894846_g1 |  |  | 1 |  |  |  |  |  |  |  |  |  |  |  | X |  |  |  |  |  |  |  |  |  |  |
| GBP5 | Hs00369472_m1 |  |  | 5 | X |  |  |  | X | X |  |  |  |  |  |  |  |  | X |  |  |  |  | X |  | X |
| GBP6 | Hs01584201_m1 |  |  | 3 |  |  |  | X |  |  |  | X |  | ‡ |  |  | X |  |  |  |  |  |  |  |  |  |
| GNG7 | Hs00192999_m1 |  |  | 1 |  |  |  |  |  |  |  |  |  | X |  |  |  |  |  |  |  |  |  |  |  |  |
| GYG1 | Hs00907542_g1 |  |  | 1 |  |  |  |  |  |  |  |  |  |  |  |  | X |  |  |  |  |  |  |  |  |  |
| GZMA | Hs00989184_m1 |  |  | 1 | X |  |  |  |  |  |  |  |  |  |  |  |  |  |  |  |  |  |  |  |  |  |
| HK3 | Hs01092850_m1 |  |  | 1 |  |  |  |  |  |  |  |  |  |  |  |  |  |  |  | X |  |  |  |  |  |  |
| ID3 | Hs00954037_g1 |  |  | 2 |  |  |  | X |  |  |  |  |  |  | X |  |  |  |  |  |  |  |  |  |  |  |
| IFI44L | Hs00915292_m1 |  |  | 1 |  |  |  |  |  |  |  |  |  |  |  |  |  |  |  | X |  |  |  |  |  |  |
| IFITM3 | Hs03057129_s1 |  |  | 2 |  |  |  |  |  | X |  |  |  |  | X |  |  |  |  |  |  |  |  |  |  |  |
| KAZN | AP7DVDD* |  |  | 1 |  |  |  |  |  |  |  |  |  |  |  |  |  |  |  |  | X |  |  |  |  |  |
| KIF1B | Hs01114512_g1 |  |  | 1 |  |  |  |  |  | X |  |  |  |  |  |  |  |  |  |  |  |  |  |  |  |  |
| KLF2 | Hs00360439_g1 |  |  | 2 |  |  |  |  | X |  |  |  |  |  |  |  |  |  |  |  |  |  |  | X |  |  |
| KLHDC8B | Hs00293902_m1 | X |  | 0 |  |  |  |  |  |  |  |  |  |  |  |  |  |  |  |  |  |  |  |  |  |  |
| KLRG1 | Hs00929964_m1 |  |  | 1 |  |  | X |  |  |  |  |  |  |  |  |  |  |  |  |  |  |  |  |  |  |  |
| LAG3 | Hs00958444_g1 |  |  | 1 |  |  |  | X |  |  |  |  |  |  |  |  |  |  |  |  |  |  |  |  |  |  |
| LHFPL2 | Hs00299613_m1 |  |  | 1 |  |  |  |  |  |  |  |  |  | X |  |  |  |  |  |  |  |  |  |  |  |  |
| LTF | Hs00158924_m1 |  |  | 1 |  |  |  |  |  |  |  |  | X |  |  |  |  |  |  |  |  |  |  |  |  |  |
| MAFB | APZTH2N* |  |  | 1 |  |  |  |  |  |  |  |  |  |  |  |  |  |  |  |  | X |  |  |  |  |  |
| MAP7D3 | Hs00226257_m1 |  |  | 1 |  |  |  |  |  |  |  |  |  |  |  |  |  |  |  |  |  |  |  |  | X |  |
| MMP9 | APEPW9P* |  |  | 1 |  |  |  |  |  | X |  |  |  |  |  |  |  |  |  |  |  |  |  |  |  |  |
| MPO | Hs00165162_m1 |  |  | 1 |  |  |  |  |  |  |  |  |  | X |  |  |  |  |  |  |  |  |  |  |  |  |
| MTRF1L | Hs01097882_g1 |  |  | 1 |  |  |  |  |  |  |  |  |  |  |  |  | X |  |  |  |  |  |  |  |  |  |
| NOD2 | Hs01550759_g1 |  |  | 1 |  |  |  |  |  | X |  |  |  |  |  |  |  |  |  |  |  |  |  |  |  |  |
| NPC2 | Hs01119244_m1 |  |  | 1 |  | X |  |  |  |  |  |  |  |  |  |  |  |  |  |  |  |  |  |  |  |  |
| OSBPL10 | Hs00215016_m1 |  |  | 1 |  |  |  |  |  |  |  |  |  |  |  |  |  |  |  |  |  | X |  |  |  |  |
| P2RY14 | Hs01848195_s1 |  |  | 1 |  |  |  |  |  |  |  |  |  |  | X |  |  |  |  |  |  |  |  |  |  |  |
| PRDM1 | Hs00153357_m1 |  |  | 1 |  |  |  |  |  |  |  | X |  |  |  |  |  |  |  |  |  |  |  |  |  |  |
| RAB13 | Hs04400188_g1 |  |  | 1 |  |  |  |  |  |  |  |  |  |  |  |  |  |  |  | X |  |  |  |  |  |  |
| Rab33A | Hs00191243_m1 |  |  | 1 |  |  |  |  |  |  |  |  | X |  |  |  |  |  |  |  |  |  |  |  |  |  |
| RABL2A | Hs00255244_m1 |  |  | 1 |  |  |  |  |  |  |  |  |  |  |  |  | X |  |  |  |  |  |  |  |  |  |
| RBBP8 | Hs01090329_m1 |  |  | 1 |  |  |  |  |  |  |  |  |  |  |  |  |  |  |  | X |  |  |  |  |  |  |
| RP11-295G20.2 | Hs01373568_m1 |  |  | 1 |  |  |  |  |  |  |  |  |  |  |  |  |  |  |  |  |  |  |  |  | X |  |
| S100A8 | Hs00374264_g1 |  |  | 1 |  |  |  |  |  |  |  |  |  | X |  |  |  |  |  |  |  |  |  |  |  |  |
| SCARF1 | Hs01092483_m1 |  |  | 1 |  |  |  |  |  |  |  |  |  |  |  |  |  |  | X |  |  |  |  |  |  |  |
| SDR39U1 | Hs01016970_g1 |  |  | 1 |  |  |  |  |  |  |  |  |  |  |  | X |  |  |  |  |  |  |  |  |  |  |
| SEPT4 | Hs00910208_g1 |  |  | 1 |  |  |  |  |  |  |  |  |  |  |  |  |  |  |  |  |  |  | X |  |  |  |
| SERPING1 | Hs00934329_m1 |  |  | 1 |  |  |  |  |  |  |  |  |  |  |  | X |  |  |  |  |  |  |  |  |  |  |
| SH2D1B | Hs01114628_m1 |  |  | 1 |  |  | X |  |  |  |  |  |  |  |  |  |  |  |  |  |  |  |  |  |  |  |
| SLPI | Hs00268206_m1 |  |  | 1 |  |  |  |  |  |  |  |  |  |  |  |  |  |  |  | X |  |  |  |  |  |  |
| SMARCD3 | Hs01088251_g1 |  |  | 3 |  |  |  |  |  |  |  |  |  | X |  |  |  |  |  | X |  |  |  |  | X |  |
| STAT1 | Hs01013996_m1 |  |  | 1 |  |  |  |  |  |  |  |  |  |  |  |  |  |  |  |  | X |  |  |  |  |  |
| STT3A | Hs00967491_m1 |  |  | 1 |  |  |  |  |  |  |  |  |  |  |  |  |  |  |  |  |  |  |  |  | X |  |
| TIMM10 | APT2DEC* |  |  | 1 |  |  |  |  |  |  |  |  |  |  |  |  |  |  |  | X |  |  |  |  |  |  |
| TMCC1 | Hs01037666_s1 |  |  | 1 |  |  |  |  |  |  |  | X |  |  |  |  |  |  |  |  |  |  |  |  |  |  |
| TNIP1 | AP47ZYZ* |  |  | 1 |  |  |  |  |  | X |  |  |  |  |  |  |  |  |  |  |  |  |  |  |  |  |
| TPPP3 | Hs00372228_g1 |  |  | 1 |  |  | X |  |  |  |  |  |  |  |  |  |  |  |  |  |  |  |  |  |  |  |
| TRMT2A | Hs01000041_g1 |  |  | 1 |  |  |  |  |  |  |  |  |  |  |  | X |  |  |  |  |  |  |  |  |  |  |
| TUBGCP6 | Hs00363509_g1 |  |  | 1 |  |  |  |  |  |  |  |  |  |  |  | X |  |  |  |  |  |  |  |  |  |  |
| UCP2 | Hs01075224_g1 |  |  | 1 |  |  |  |  |  |  |  |  |  |  |  |  |  |  |  |  |  |  |  |  | X |  |
| VAMP5 | Hs01105383_g1 |  |  | 1 |  |  |  |  |  |  |  |  |  | X |  |  |  |  |  |  |  |  |  |  |  |  |
| WARS-j1 | Hs00998737_m1 | X |  | 0 |  |  |  |  |  |  |  |  |  |  |  |  |  |  |  |  |  |  |  |  |  |  |
| WARS-j2 | APAAFHX* | X |  | 0 |  |  |  |  |  |  |  |  |  |  |  |  |  |  |  |  |  |  |  |  |  |  |
| ZDHHC19 | Hs00376118_m1 |  |  | 1 |  |  |  | X |  |  |  |  |  |  |  |  |  |  |  |  |  |  |  |  |  |  |
| ZFYVE9 | Hs01024382_m1 |  |  | 1 |  |  | # |  |  |  |  |  |  |  |  |  |  |  |  |  |  |  |  |  |  |  |
| ZNF296 | Hs00377132_m1 |  |  | 2 |  |  |  |  |  |  | X |  |  | X |  |  |  |  |  |  |  |  |  |  |  |  |

Signatures are named by first author and number of transcripts included in the model (e.g. Author11). Numbers in brackets indicate the original number of transcripts in the published model. Some signatures have a reduced number of transcripts when translated to RT-qPCR due to duplicate transcript symbols (IDs), high-primer probe failure rate (**poor amplification efficiency), or where transcript sequences from an original discovery cohorts could not be mapped to a more recent reference transcriptome.

* Custom designed primer-probe assays; available on request to corresponding author.

\# One transcripts (ZFYVE9) was excluded from the Duffy5 signature due to a high primer-probe assay failure rate.

† The CERKL1 primer-probe assay failed during panel optimisation and was removed from the Duffy10 signature.

‡ Three transcripts (C1QB, C1QC, and GBP6) were excluded from the Kaforou27 signature due to a high primer-probe assay failure rate. Unique primer-probe assays could not be designed for both of the FCGR1B transcript variants; only one FCGR1B primer-probe was included in the Kaforou22 model. We were unable to design a primer-probe for LOC728744; all information had been withdrawn from both the NCBI and RefSeq databases.

Xpert HR=Xpert Host-Response.

## Table S2. Transcriptomic signatures on the panel that were reparameterised.

| **Signature** | **Reference** | **Original gene expression measurement** | **Model used** | **HIV-negative training and test set (combined)**  **AUC (95% CI)** | **HIV-positive validation set**  **AUC (95% CI)** |
| --- | --- | --- | --- | --- | --- |
| **Da Costa3** | *Tuberculosis* (2015)^4^ | RT-qPCR | Random forest | 0.98 (0.93−1) | 0.96 (0.93−1) |
| **Duffy9** | *PLoS One* (2019)^5^ | Meta-analysis of microarray data | Multinomial random forest | TB vs LTBI: 1 (1−1)* | |
| **Francisco2** | *J Infect* (2017)^6^ | RT-qPCR | Random forest | 0.99 (0.98−1) | 0.83 (0.74−0.93) |
| **Gjøen7** | *Sci Rep* (2017)^7^ | Dual-color-Reverse-Transcriptase-Multiplex-Ligation-dependent-Probe-Amplification (dc-RT MLPA) | LASSO regression | 0.98 (0.94−1) | 0.78 (0.67−0.88) |
| **Jacobsen3** | *J Mol Med* (2007)^8^ | RT-qPCR | Linear discriminant analysis | 0.99 (0.99−1) | 0.95 (0.89−1) |
| **Roe3** | *Clin Infect Dis* (2020)^9^ | RNA sequencing | Support vector machines (linear kernel) | 0.96 (0.89−1) | 0.89 (0.80−0.97) |
| **Sambarey10** | *EBioMedicine* (2017)^10^ | RNA sequencing | Linear discriminant analysis | 0.99 (0.96−1) | 0.95 (0.90−1) |
| **Satproedprai7** | *Genes Immun* (2015)^11^ | RT-qPCR | LASSO regression | 0.99 (0.98−1) | 0.95 (0.90 −1) |

* HIV-positive samples required to parameterise the multinomial model. No independent validation set available.

## Table S3. Target Product Profile for TB treatment biomarkers.

| **Timing of test** | **Use Case** | **Notes** | **Sensitivity** | | **Specificity** | |
| --- | --- | --- | --- | --- | --- | --- |
|  |  |  | **Minimum** | **Optimum** | **Minimum** | **Optimum** |
| **Baseline/ diagnosis**  **Treatment initiation** | Identify those who require more intensive treatment regimen OR shorter regimen | To avoid undertreatment of those with higher risk  (e.g., more severe disease) | **>90%** | >95% | **>70%** | >80% |
| **During treatment** | Identify those at risk of poor outcome OR for shorter regimen | Identify those who do not adequately respond  Adherence support | **>75%** | >90% | **>80%** | >90% |
| **End of treatment** | Identify those with poor outcome | Prolong treatment | **>80%** | >95% | **>90%** | >95% |

Reported by Gupta-Wright et al. (2023)^28^ and WHO (2023)^29^

## Table S4A. Performance (area under the curve) of transcriptomic signatures measured at baseline, month 2, and end of treatment (month 6) for predicting TB recurrence after completion of treatment.

| **Signature** | **Baseline controls** | **Baseline cases** | **Baseline**  **AUC (95% CI)** | **Month 2 controls** | **Month 2 cases** | **Month 2**  **AUC (95% CI)** | **End of treatment controls** | **End of treatment cases** | **End of treatment**  **AUC (95% CI)** |
| --- | --- | --- | --- | --- | --- | --- | --- | --- | --- |
| **da Costa3** | 259 | 9 | 0.71 (0.60–0.82) | 261 | 9 | 0.80 (0.67–0.94) | 259 | 9 | 0.84 (0.70–0.98) |
| **de Araujo1** | 259 | 9 | 0.91 (0.83–0.98) | 261 | 9 | 0.88 (0.79–0.98) | 259 | 9 | 0.77 (0.62–0.92) |
| **Duffy4** | 246 | 9 | 0.83 (0.67–0.99) | 257 | 8 | 0.77 (0.60–0.93) | 256 | 9 | 0.64 (0.47–0.80) |
| **Duffy9** | 249 | 9 | 0.78 (0.63–0.94) | 247 | 8 | 0.85 (0.71–0.98) | 236 | 9 | 0.73 (0.57–0.89) |
| **Francisco2** | 248 | 7 | 0.80 (0.72–0.89) | 252 | 7 | 0.78 (0.54–1.00) | 248 | 9 | 0.82 (0.66–0.98) |
| **Gjoen7** | 259 | 9 | 0.82 (0.71–0.92) | 258 | 9 | 0.84 (0.68–1.00) | 259 | 9 | 0.89 (0.82–0.97) |
| **Gliddon3** | 243 | 6 | 0.91 (0.83–0.99) | 243 | 6 | 0.84 (0.72–0.97) | 234 | 8 | 0.71 (0.49–0.93) |
| **Gliddon4** | 254 | 9 | 0.85 (0.76–0.95) | 240 | 9 | 0.78 (0.58–0.98) | 220 | 9 | 0.59 (0.35–0.83) |
| **Jacobsen3** | 217 | 7 | 0.75 (0.62–0.87) | 209 | 7 | 0.81 (0.64–0.98) | 200 | 9 | 0.81 (0.64–0.99) |
| **Kaforou22** | 227 | 6 | 0.91 (0.83–0.99) | 215 | 6 | 0.87 (0.71–1.00) | 182 | 6 | 0.75 (0.49–1.00) |
| **Maertzdorf4** | 251 | 8 | 0.82 (0.64–0.99) | 254 | 8 | 0.80 (0.58–1.00) | 250 | 9 | 0.76 (0.55–0.97) |
| **Penn-Nicholson6** | 258 | 9 | 0.78 (0.60–0.96) | 260 | 8 | 0.79 (0.61–0.97) | 258 | 9 | 0.77 (0.58–0.97) |
| **Rajan5** | 215 | 7 | 0.72 (0.57–0.88) | 193 | 8 | 0.73 (0.53–0.93) | 175 | 9 | 0.67 (0.49–0.85) |
| **Roe1** | 258 | 9 | 0.78 (0.68–0.88) | 258 | 9 | 0.82 (0.69–0.95) | 255 | 9 | 0.73 (0.52–0.94) |
| **Roe3** | 257 | 9 | 0.85 (0.74–0.95) | 258 | 9 | 0.82 (0.66–0.99) | 251 | 9 | 0.83 (0.70–0.95) |
| **Sambarey10** | 254 | 9 | 0.76 (0.67–0.86) | 255 | 8 | 0.82 (0.67–0.98) | 254 | 9 | 0.77 (0.57–0.97) |
| **Satproedprai7** | 254 | 9 | 0.88 (0.79–0.97) | 249 | 9 | 0.86 (0.72–1.00) | 244 | 9 | 0.82 (0.65–0.99) |
| **Suliman2** | 149 | 2 | 0.76 (0.35–1.00) | 150 | 4 | 0.80 (0.61–1.00) | 163 | 8 | 0.67 (0.47–0.87) |
| **Suliman4** | 249 | 9 | 0.71 (0.51–0.90) | 252 | 8 | 0.84 (0.78–0.91) | 247 | 9 | 0.64 (0.47–0.82) |
| **Sweeney3** | 248 | 7 | 0.82 (0.70–0.94) | 252 | 7 | 0.78 (0.59–0.97) | 246 | 9 | 0.73 (0.54–0.93) |
| **Thompson5** | 257 | 9 | 0.79 (0.68–0.89) | 261 | 8 | 0.79 (0.65–0.94) | 258 | 9 | 0.42 (0.19–0.65) |
| **Xpert HR** | 259 | 9 | 0.80 (0.66–0.94) | 261 | 9 | 0.84 (0.72–0.95) | 257 | 9 | 0.84 (0.71–0.96) |

AUC=area under the curve. CI=confidence interval. Xpert HR=Xpert Host-Response.

## Table S4B. Sensitivity analysis: Performance of transcriptomic signatures measured at baseline, month 2, and end of treatment (month 6), versus covariate-adjusted signature models, for predicting TB recurrence after completion of treatment.

| **Signature** | **Baseline covariate-adjusted model**  **ΔAUC (95% CI; p-value)** | **Month 2 covariate-adjusted model**  **ΔAUC (95% CI; p-value)** | **End of treatment covariate-adjusted model**  **ΔAUC (95% CI; p-value)** |
| --- | --- | --- | --- |
| **da Costa3** | 0.13 (-0.02–0.28; 0.089) | -0.02 (-0.14–0.10; 0.74) | 0.02 (-0.04–0.07; 0.62) |
| **de Araujo1** | 0.02 (-0.02–0.06; 0.32) | 0.00 (-0.03–0.03; 0.88) | 0.05 (-0.07–0.16; 0.43) |
| **Duffy4** | 0.03 (-0.04–0.11; 0.39) | 0.11 (-0.02–0.23; 0.091) | 0.12 (-0.09–0.34; 0.25) |
| **Duffy9** | 0.03 (-0.07–0.14; 0.53) | 0.01 (-0.01–0.03; 0.56) | 0.07 (-0.08–0.22; 0.34) |
| **Francisco2** | **0.12 (0.05–0.20; 0.0014)** | -0.01 (-0.10–0.09; 0.91) | -0.01 (-0.09–0.08; 0.85) |
| **Gjoen7** | 0.04 (-0.05–0.14; 0.38) | 0.00 (-0.05–0.05; 0.99) | 0.00 (-0.02–0.02; 0.89) |
| **Gliddon3** | 0.01 (-0.03–0.04; 0.74) | 0.02 (-0.03–0.06; 0.47) | 0.10 (-0.06–0.26; 0.24) |
| **Gliddon4** | 0.02 (-0.03–0.07; 0.44) | 0.02 (-0.04–0.07; 0.54) | 0.14 (-0.07–0.36; 0.19) |
| **Jacobsen3** | **0.16 (0.02–0.31; 0.026)** | -0.04 (-0.18–0.09; 0.53) | 0.03 (-0.03–0.08; 0.30) |
| **Kaforou22** | 0.01 (-0.02–0.04; 0.38) | 0.01 (-0.02–0.04; 0.56) | 0.15 (-0.05–0.35; 0.13) |
| **Maertzdorf4** | 0.04 (-0.06–0.15; 0.41) | 0.00 (-0.01–0.01; 0.71) | 0.03 (-0.05–0.11; 0.47) |
| **Penn-Nicholson6** | 0.05 (-0.08–0.19; 0.42) | 0.00 (-0.05–0.06; 0.87) | 0.03 (-0.04–0.11; 0.40) |
| **Rajan5** | **0.15 (0.05–0.25; 0.0041)** | 0.03 (-0.09–0.14; 0.65) | 0.04 (-0.09–0.17; 0.54) |
| **Roe1** | 0.07 (-0.03–0.17; 0.16) | 0.00 (-0.04–0.04; 0.88) | 0.05 (-0.05–0.15; 0.36) |
| **Roe3** | 0.04 (-0.04–0.13; 0.30) | 0.01 (-0.04–0.06; 0.75) | 0.01 (-0.04–0.06; 0.65) |
| **Sambarey10** | 0.09 (-0.03–0.21; 0.14) | -0.03 (-0.15–0.08; 0.57) | 0.05 (-0.02–0.13; 0.19) |
| **Satproedprai7** | 0.03 (-0.04–0.10; 0.35) | 0.00 (-0.05–0.05; 0.97) | 0.01 (-0.03–0.06; 0.51) |
| **Suliman2** | 0.07 (-0.28–0.41; 0.70) | 0.08 (-0.04–0.19; 0.18) | 0.09 (-0.09–0.27; 0.34) |
| **Suliman4** | 0.08 (-0.09–0.26; 0.34) | 0.02 (-0.04–0.07; 0.59) | 0.11 (-0.08–0.31; 0.26) |
| **Sweeney3** | 0.08 (-0.01–0.17; 0.069) | 0.05 (-0.01–0.12; 0.12) | 0.04 (-0.07–0.15; 0.52) |
| **Thompson5** | 0.06 (-0.04–0.17; 0.24) | 0.03 (-0.05–0.12; 0.43) | **0.35 (0.02–0.69; 0.038)** |
| **Xpert HR** | 0.05 (-0.06–0.16; 0.36) | 0.02 (-0.02–0.06; 0.41) | 0.01 (-0.02–0.05; 0.47) |

AUC=area under the curve. CI=confidence interval. Xpert HR=Xpert Host-Response.

ΔAUC is the difference in AUC between the covariate-adjusted signature model (signature + age + sex + HIV) minus the AUC for the base signature model (signature only).

## Table S5. Sensitivity, specificity, positive predictive value, and negative predictive value of transcriptomic signatures measured at baseline, month 2, and end of treatment (month 6) for predicting TB recurrence after completion of treatment.

| **Signature** | **Baseline sensitivity, %**  **(95% CI)** | **Baseline specificity, %**  **(95% CI)** | **Month 2 sensitivity, %**  **(95% CI)** | **Month 2**  **specificity, %**  **(95% CI)** | **End of treatment**  **sensitivity, %**  **(95% CI)** | **End of treatment**  **specificity, %**  **(95% CI)** |
| --- | --- | --- | --- | --- | --- | --- |
| **WHO Target Product Profile for TB treatment biomarkers** | Minimum: 90%  Optimum: 95% | **Minimum: 70% ***  Optimum: 80% | Minimum: 75%  Optimum: 90% | **Minimum: 80% ***  Optimum: 90% | Minimum: 80%  Optimum: 95% | **Minimum: 90% ***  Optimum: 95% |
| **da Costa3** | 55.6 (26.7– 81.1) | 69.9 (64.0–75.1) | 55.6 (26.7– 81.1) | 79.7 (74.4–84.1) | 55.6 (26.7– 81.1) | 89.6 (85.3–92.7) |
| **de Araujo1** | 88.9 (56.5– 98.0) | 69.9 (64.0–75.1) | 88.9 (56.5– 98.0) | 79.7 (74.4–84.1) | 33.3 (12.1– 64.6) | 89.6 (85.3–92.7) |
| **Duffy4** | 88.9 (56.5– 98.0) | 69.5 (63.5–74.9) | 62.5 (30.6– 86.3) | 79.8 (74.4–84.2) | 22.2 ( 6.3– 54.7) | 89.8 (85.5–93.0) |
| **Duffy9** | 77.8 (45.3– 93.7) | 69.9 (63.9–75.2) | 87.5 (52.9– 97.8) | 79.8 (74.3–84.3) | 33.3 (12.1– 64.6) | 89.8 (85.3–93.1) |
| **Francisco2** | 85.7 (48.7– 97.4) | 70.6 (64.6–75.9) | 71.4 (35.9– 91.8) | 80.6 (75.2–85.0) | 66.7 (35.4– 87.9) | 89.5 (85.1–92.7) |
| **Gjoen7** | 88.9 (56.5– 98.0) | 69.9 (64.0–75.1) | 77.8 (45.3– 93.7) | 79.8 (74.5–84.3) | 66.7 (35.4– 87.9) | 89.6 (85.3–92.7) |
| **Gliddon3** | 100.0 (61.0–100.0) | 69.5 (63.5–75.0) | 66.7 (30.0– 90.3) | 79.8 (74.3–84.4) | 37.5 (13.7– 69.4) | 89.7 (85.2–93.0) |
| **Gliddon4** | 77.8 (45.3– 93.7) | 69.7 (63.8–75.0) | 77.8 (45.3– 93.7) | 79.6 (74.0–84.2) | 33.3 (12.1– 64.6) | 89.5 (84.8–92.9) |
| **Jacobsen3** | 42.9 (15.8– 75.0) | 69.6 (63.2–75.3) | 71.4 (35.9– 91.8) | 79.9 (74.0–84.8) | 44.4 (18.9– 73.3) | 89.5 (84.5–93.0) |
| **Kaforou22** | 100.0 (61.0–100.0) | 69.6 (63.3–75.2) | 83.3 (43.6– 97.0) | 79.5 (73.6–84.4) | 50.0 (18.8– 81.2) | 89.6 (84.3–93.2) |
| **Maertzdorf4** | 75.0 (40.9– 92.9) | 69.7 (63.8–75.1) | 87.5 (52.9– 97.8) | 79.5 (74.1–84.0) | 44.4 (18.9– 73.3) | 89.6 (85.2–92.8) |
| **Penn-Nicholson6** | 77.8 (45.3– 93.7) | 69.8 (63.9–75.0) | 75.0 (40.9– 92.9) | 79.6 (74.3–84.1) | 44.4 (18.9– 73.3) | 89.5 (85.2–92.7) |
| **Rajan5** | 57.1 (25.0– 84.2) | 69.8 (63.3–75.5) | 50.0 (21.5– 78.5) | 79.8 (73.6–84.9) | 22.2 ( 6.3– 54.7) | 89.7 (84.3–93.4) |
| **Roe1** | 77.8 (45.3– 93.7) | 69.8 (63.9–75.0) | 55.6 (26.7– 81.1) | 79.8 (74.5–84.3) | 44.4 (18.9– 73.3) | 89.8 (85.5–92.9) |
| **Roe3** | 77.8 (45.3– 93.7) | 69.6 (63.8–74.9) | 77.8 (45.3– 93.7) | 79.8 (74.5–84.3) | 55.6 (26.7– 81.1) | 89.6 (85.3–92.8) |
| **Sambarey10** | 66.7 (35.4– 87.9) | 69.7 (63.8–75.0) | 75.0 (40.9– 92.9) | 79.6 (74.2–84.1) | 55.6 (26.7– 81.1) | 89.8 (85.4–92.9) |
| **Satproedprai7** | 88.9 (56.5– 98.0) | 69.7 (63.8–75.0) | 88.9 (56.5– 98.0) | 79.5 (74.1–84.1) | 44.4 (18.9– 73.3) | 89.8 (85.3–93.0) |
| **Suliman2** | 50.0 ( 9.5– 90.5) | 69.8 (62.0–76.6) | 75.0 (30.1– 95.4) | 80.0 (72.9–85.6) | 12.5 ( 2.2– 47.1) | 89.6 (83.9–93.4) |
| **Suliman4** | 66.7 (35.4– 87.9) | 69.9 (63.9–75.2) | 75.0 (40.9– 92.9) | 79.8 (74.4–84.3) | 11.1 ( 2.0– 43.5) | 89.9 (85.5–93.0) |
| **Sweeney3** | 71.4 (35.9– 91.8) | 69.8 (63.8–75.1) | 71.4 (35.9– 91.8) | 79.8 (74.4–84.3) | 22.2 ( 6.3– 54.7) | 89.8 (85.4–93.0) |
| **Thompson5** | 66.7 (35.4– 87.9) | 69.6 (63.8–74.9) | 62.5 (30.6– 86.3) | 79.7 (74.4–84.1) | 11.1 ( 2.0– 43.5) | 89.5 (85.2–92.7) |
| **Xpert HR** | 77.8 (45.3– 93.7) | 69.9 (64.0–75.1) | 88.9 (56.5– 98.0) | 79.7 (74.4–84.1) | 55.6 (26.7– 81.1) | 89.9 (85.6–93.0) |

* Signatures benchmarked at 70%, 80%, and 90% specificity at baseline, month 2, and end of treatment, respectively.

CI=confidence interval. Xpert HR=Xpert Host-Response.

## Table S6A. Performance (area under the curve) of transcriptomic signatures measured at baseline, month 2, and end of treatment (month 6) for predicting TB treatment failure.

| **Signature** | **Baseline controls** | **Baseline cases** | **Baseline**  **AUC (95% CI)** | **Month 2 controls** | **Month 2 cases** | **Month 2**  **AUC (95% CI)** | **End of treatment controls** | **End of treatment cases** | **End of treatment**  **AUC (95% CI)** |
| --- | --- | --- | --- | --- | --- | --- | --- | --- | --- |
| **da Costa3** | 259 | 32 | 0.64 (0.54–0.73) | 261 | 31 | 0.55 (0.45–0.65) | 259 | 28 | 0.60 (0.48–0.71) |
| **de Araujo1** | 259 | 32 | 0.69 (0.60–0.77) | 261 | 31 | 0.56 (0.46–0.66) | 259 | 28 | 0.62 (0.51–0.73) |
| **Duffy4** | 246 | 28 | 0.62 (0.51–0.72) | 257 | 29 | 0.63 (0.52–0.73) | 256 | 27 | 0.57 (0.46–0.68) |
| **Duffy9** | 249 | 31 | 0.57 (0.46–0.69) | 247 | 28 | 0.53 (0.41–0.64) | 236 | 24 | 0.62 (0.51–0.72) |
| **Francisco2** | 248 | 26 | 0.68 (0.57–0.80) | 252 | 28 | 0.51 (0.40–0.62) | 248 | 26 | 0.58 (0.46–0.70) |
| **Gjoen7** | 259 | 32 | 0.66 (0.56–0.76) | 258 | 31 | 0.53 (0.42–0.63) | 259 | 28 | 0.47 (0.36–0.58) |
| **Gliddon3** | 243 | 26 | 0.56 (0.44–0.68) | 243 | 29 | 0.55 (0.43–0.67) | 234 | 25 | 0.45 (0.33–0.57) |
| **Gliddon4** | 254 | 31 | 0.58 (0.47–0.69) | 240 | 29 | 0.53 (0.42–0.63) | 220 | 22 | 0.52 (0.37–0.68) |
| **Jacobsen3** | 217 | 26 | 0.61 (0.50–0.72) | 209 | 29 | 0.57 (0.46–0.68) | 200 | 26 | 0.59 (0.47–0.72) |
| **Kaforou22** | 227 | 22 | 0.49 (0.38–0.61) | 215 | 24 | 0.54 (0.41–0.67) | 182 | 16 | 0.60 (0.41–0.78) |
| **Maertzdorf4** | 251 | 32 | 0.58 (0.47–0.68) | 254 | 29 | 0.49 (0.38–0.60) | 250 | 27 | 0.53 (0.43–0.64) |
| **Penn-Nicholson6** | 258 | 32 | 0.63 (0.53–0.74) | 260 | 31 | 0.55 (0.45–0.65) | 258 | 28 | 0.59 (0.49–0.70) |
| **Rajan5** | 215 | 26 | 0.51 (0.38–0.63) | 193 | 28 | 0.62 (0.50–0.74) | 175 | 22 | 0.50 (0.35–0.65) |
| **Roe1** | 258 | 32 | 0.62 (0.53–0.71) | 258 | 31 | 0.52 (0.42–0.63) | 255 | 26 | 0.57 (0.45–0.69) |
| **Roe3** | 257 | 32 | 0.69 (0.59–0.78) | 258 | 31 | 0.50 (0.40–0.60) | 251 | 26 | 0.52 (0.39–0.64) |
| **Sambarey10** | 254 | 31 | 0.64 (0.54–0.74) | 255 | 31 | 0.55 (0.45–0.66) | 254 | 27 | 0.61 (0.49–0.73) |
| **Satproedprai7** | 254 | 32 | 0.64 (0.54–0.73) | 249 | 28 | 0.59 (0.48–0.70) | 244 | 27 | 0.62 (0.50–0.73) |
| **Suliman2** | 149 | 17 | 0.55 (0.41–0.69) | 150 | 18 | 0.52 (0.37–0.66) | 163 | 20 | 0.65 (0.53–0.77) |
| **Suliman4** | 249 | 31 | 0.50 (0.39–0.61) | 252 | 30 | 0.51 (0.40–0.62) | 247 | 26 | 0.53 (0.41–0.66) |
| **Sweeney3** | 248 | 26 | 0.66 (0.55–0.76) | 252 | 28 | 0.52 (0.41–0.62) | 246 | 26 | 0.53 (0.41–0.64) |
| **Thompson5** | 257 | 31 | 0.66 (0.56–0.76) | 261 | 31 | 0.63 (0.53–0.73) | 258 | 28 | 0.66 (0.56–0.77) |
| **Xpert HR** | 259 | 32 | 0.57 (0.47–0.66) | 261 | 31 | 0.50 (0.40–0.61) | 257 | 28 | 0.52 (0.39–0.65) |

AUC=area under the curve. CI=confidence interval. Xpert HR=Xpert Host-Response.

## Table S6B. Sensitivity analysis: Performance of transcriptomic signatures measured at baseline, month 2, and end of treatment (month 6), versus covariate-adjusted signature models, for predicting TB treatment failure.

| **Signature** | **Baseline covariate-adjusted model**  **ΔAUC (95% CI; p-value)** | **Month 2 covariate-adjusted model**  **ΔAUC (95% CI; p-value)** | **End of treatment covariate-adjusted model**  **ΔAUC (95% CI; p-value)** |
| --- | --- | --- | --- |
| **da Costa3** | 0.03 (-0.07–0.13; 0.61) | **0.10 (0.00–0.20; 0.048)** | 0.07 (-0.02–0.16; 0.14) |
| **de Araujo1** | 0.05 (-0.01–0.11; 0.091) | 0.09 (-0.02–0.20; 0.12) | 0.05 (-0.04–0.14; 0.26) |
| **Duffy4** | 0.05 (-0.02–0.12; 0.13) | 0.06 (-0.02–0.14; 0.12) | 0.06 (-0.06–0.19; 0.33) |
| **Duffy9** | 0.06 (-0.04–0.16; 0.26) | 0.12 (-0.01–0.24; 0.063) | 0.08 (-0.05–0.21; 0.25) |
| **Francisco2** | -0.01 (-0.11–0.10; 0.92) | 0.10 (-0.04–0.24; 0.14) | 0.05 (-0.04–0.15; 0.26) |
| **Gjoen7** | 0.04 (-0.04–0.13; 0.34) | **0.13 (0.01–0.24; 0.039)** | 0.15 (-0.03–0.34; 0.10) |
| **Gliddon3** | 0.07 (-0.06–0.19; 0.29) | **0.12 (0.00–0.23; 0.047)** | **0.25 (0.08–0.43; 0.0038)** |
| **Gliddon4** | 0.06 (-0.03–0.16; 0.18) | 0.12 (-0.01–0.26; 0.081) | 0.13 (-0.01–0.26; 0.069) |
| **Jacobsen3** | 0.05 (-0.05–0.15; 0.35) | 0.09 (-0.03–0.20; 0.15) | 0.09 (-0.03–0.21; 0.14) |
| **Kaforou22** | 0.16 (-0.01–0.33; 0.059) | **0.13 (0.00–0.25; 0.044)** | 0.13 (0.00–0.26; 0.058) |
| **Maertzdorf4** | 0.08 (-0.02–0.17; 0.11) | 0.15 (-0.02–0.33; 0.088) | 0.08 (-0.08–0.23; 0.34) |
| **Penn-Nicholson6** | 0.05 (-0.04–0.14; 0.27) | 0.10 (0.00–0.21; 0.061) | 0.07 (-0.03–0.17; 0.16) |
| **Rajan5** | 0.08 (-0.09–0.25; 0.34) | 0.03 (-0.06–0.12; 0.53) | 0.14 (-0.02–0.29; 0.090) |
| **Roe1** | 0.08 (0.00–0.16; 0.062) | 0.12 (0.00–0.25; 0.056) | 0.08 (-0.03–0.20; 0.15) |
| **Roe3** | 0.03 (-0.03–0.09; 0.30) | 0.12 (-0.02–0.26; 0.10) | 0.12 (-0.02–0.27; 0.089) |
| **Sambarey10** | 0.06 (-0.03–0.16; 0.16) | **0.11 (0.00–0.22; 0.045)** | 0.07 (-0.04–0.17; 0.21) |
| **Satproedprai7** | 0.05 (-0.02–0.12; 0.13) | 0.07 (-0.02–0.16; 0.15) | 0.09 (-0.02–0.19; 0.11) |
| **Suliman2** | 0.10 (-0.04–0.24; 0.15) | 0.08 (-0.15–0.31; 0.49) | 0.07 (-0.06–0.20; 0.28) |
| **Suliman4** | 0.13 (-0.02–0.27; 0.087) | 0.11 (-0.02–0.24; 0.10) | 0.10 (-0.04–0.23; 0.17) |
| **Sweeney3** | 0.03 (-0.04–0.10; 0.34) | 0.09 (-0.04–0.23; 0.17) | 0.07 (-0.09–0.22; 0.40) |
| **Thompson5** | 0.04 (-0.02–0.09; 0.18) | 0.07 (-0.02–0.15; 0.11) | 0.03 (-0.04–0.11; 0.40) |
| **Xpert HR** | 0.08 (-0.01–0.17; 0.087) | 0.11 (-0.03–0.26; 0.13) | 0.10 (-0.09–0.30; 0.31) |

AUC=area under the curve. CI=confidence interval. Xpert HR=Xpert Host-Response.

ΔAUC is the difference in AUC between the covariate-adjusted signature model (signature + age + sex + HIV) minus the AUC for the base signature model (signature only).

## Table S7. Sensitivity, specificity, positive predictive value, and negative predictive value of transcriptomic signatures measured at baseline, month 2, and end of treatment (month 6) for predicting TB treatment failure.

| **Signature** | **Baseline sensitivity, %**  **(95% CI)** | **Baseline specificity, %**  **(95% CI)** | **Month 2 sensitivity, %**  **(95% CI)** | **Month 2**  **specificity, %**  **(95% CI)** | **End of treatment**  **sensitivity, %**  **(95% CI)** | **End of treatment**  **specificity, %**  **(95% CI)** |
| --- | --- | --- | --- | --- | --- | --- |
| **WHO Target Product Profile for TB treatment biomarkers** | Minimum: 90%  Optimum: 95% | **Minimum: 70% ***  Optimum: 80% | Minimum: 75%  Optimum: 90% | **Minimum: 80% ***  Optimum: 90% | Minimum: 80%  Optimum: 95% | **Minimum: 90% ***  Optimum: 95% |
| **da Costa3** | 40.6 (25.5–57.7) | 69.9 (64.0–75.1) | 19.4 ( 9.2–36.3) | 79.7 (74.4–84.1) | 17.9 ( 7.9–35.6) | 89.6 (85.3–92.7) |
| **de Araujo1** | 50.0 (33.6–66.4) | 69.9 (64.0–75.1) | 12.9 ( 5.1–28.9) | 79.7 (74.4–84.1) | 14.3 ( 5.7–31.5) | 89.6 (85.3–92.7) |
| **Duffy4** | 53.6 (35.8–70.5) | 69.5 (63.5–74.9) | 37.9 (22.7–56.0) | 79.8 (74.4–84.2) | 14.8 ( 5.9–32.5) | 89.8 (85.5–93.0) |
| **Duffy9** | 45.2 (29.2–62.2) | 69.9 (63.9–75.2) | 25.0 (12.7–43.4) | 79.8 (74.3–84.3) | 8.3 ( 2.3–25.8) | 89.8 (85.3–93.1) |
| **Francisco2** | 57.7 (38.9–74.5) | 70.6 (64.6–75.9) | 14.3 ( 5.7–31.5) | 80.6 (75.2–85.0) | 23.1 (11.0–42.1) | 89.5 (85.1–92.7) |
| **Gjoen7** | 53.1 (36.4–69.1) | 69.9 (64.0–75.1) | 19.4 ( 9.2–36.3) | 79.8 (74.5–84.3) | 3.6 ( 0.6–17.7) | 89.6 (85.3–92.7) |
| **Gliddon3** | 38.5 (22.4–57.5) | 69.5 (63.5–75.0) | 20.7 ( 9.8–38.4) | 79.8 (74.3–84.4) | 8.0 ( 2.2–25.0) | 89.7 (85.2–93.0) |
| **Gliddon4** | 38.7 (23.7–56.2) | 69.7 (63.8–75.0) | 20.7 ( 9.8–38.4) | 79.6 (74.0–84.2) | 22.7 (10.1–43.4) | 89.5 (84.8–92.9) |
| **Jacobsen3** | 42.3 (25.5–61.1) | 69.6 (63.2–75.3) | 27.6 (14.7–45.7) | 79.9 (74.0–84.8) | 23.1 (11.0–42.1) | 89.5 (84.5–93.0) |
| **Kaforou22** | 18.2 ( 7.3–38.5) | 69.6 (63.3–75.2) | 25.0 (12.0–44.9) | 79.5 (73.6–84.4) | 31.2 (14.2–55.6) | 89.6 (84.3–93.2) |
| **Maertzdorf4** | 46.9 (30.9–63.6) | 69.7 (63.8–75.1) | 24.1 (12.2–42.1) | 79.5 (74.1–84.0) | 7.4 ( 2.1–23.4) | 89.6 (85.2–92.8) |
| **Penn-Nicholson6** | 50.0 (33.6–66.4) | 69.8 (63.9–75.0) | 19.4 ( 9.2–36.3) | 79.6 (74.3–84.1) | 17.9 ( 7.9–35.6) | 89.5 (85.2–92.7) |
| **Rajan5** | 30.8 (16.5–50.0) | 69.8 (63.3–75.5) | 35.7 (20.7–54.2) | 79.8 (73.6–84.9) | 13.6 ( 4.7–33.3) | 89.7 (84.3–93.4) |
| **Roe1** | 40.6 (25.5–57.7) | 69.8 (63.9–75.0) | 19.4 ( 9.2–36.3) | 79.8 (74.5–84.3) | 15.4 ( 6.2–33.5) | 89.8 (85.5–92.9) |
| **Roe3** | 50.0 (33.6–66.4) | 69.6 (63.8–74.9) | 12.9 ( 5.1–28.9) | 79.8 (74.5–84.3) | 11.5 ( 4.0–29.0) | 89.6 (85.3–92.8) |
| **Sambarey10** | 48.4 (32.0–65.2) | 69.7 (63.8–75.0) | 29.0 (16.1–46.6) | 79.6 (74.2–84.1) | 22.2 (10.6–40.8) | 89.8 (85.4–92.9) |
| **Satproedprai7** | 43.8 (28.2–60.7) | 69.7 (63.8–75.0) | 25.0 (12.7–43.4) | 79.5 (74.1–84.1) | 18.5 ( 8.2–36.7) | 89.8 (85.3–93.0) |
| **Suliman2** | 35.3 (17.3–58.7) | 69.8 (62.0–76.6) | 27.8 (12.5–50.9) | 80.0 (72.9–85.6) | 10.0 ( 2.8–30.1) | 89.6 (83.9–93.4) |
| **Suliman4** | 41.9 (26.4–59.2) | 69.9 (63.9–75.2) | 16.7 ( 7.3–33.6) | 79.8 (74.4–84.3) | 19.2 ( 8.5–37.9) | 89.9 (85.5–93.0) |
| **Sweeney3** | 46.2 (28.8–64.5) | 69.8 (63.8–75.1) | 17.9 ( 7.9–35.6) | 79.8 (74.4–84.3) | 7.7 ( 2.1–24.1) | 89.8 (85.4–93.0) |
| **Thompson5** | 58.1 (40.8–73.6) | 69.6 (63.8–74.9) | 25.8 (13.7–43.2) | 79.7 (74.4–84.1) | 17.9 ( 7.9–35.6) | 89.5 (85.2–92.7) |
| **Xpert HR** | 25.0 (13.3–42.1) | 69.9 (64.0–75.1) | 16.1 ( 7.1–32.6) | 79.7 (74.4–84.1) | 17.9 ( 7.9–35.6) | 89.9 (85.6–93.0) |

* Signatures benchmarked at 70%, 80%, and 90% specificity at baseline, month 2, and end of treatment, respectively.

CI=confidence interval. Xpert HR=Xpert Host-Response.

## Table S8A. Performance (area under the curve) of transcriptomic signatures measured at baseline for predicting death due to TB or unknown cause.

| **Signature** | **Baseline controls** | **Baseline cases** | **Baseline**  **AUC (95% CI)** |
| --- | --- | --- | --- |
| **da Costa3** | 259 | 20 | 0.52 (0.39–0.65) |
| **de Araujo1** | 259 | 20 | 0.54 (0.39–0.68) |
| **Duffy4** | 246 | 19 | 0.81 (0.70–0.92) |
| **Duffy9** | 249 | 19 | 0.72 (0.62–0.81) |
| **Francisco2** | 248 | 20 | 0.56 (0.42–0.70) |
| **Gjoen7** | 259 | 20 | 0.54 (0.39–0.68) |
| **Gliddon3** | 243 | 17 | 0.71 (0.61–0.81) |
| **Gliddon4** | 254 | 18 | 0.76 (0.64–0.87) |
| **Jacobsen3** | 217 | 13 | 0.73 (0.64–0.82) |
| **Kaforou22** | 227 | 16 | 0.73 (0.62–0.85) |
| **Maertzdorf4** | 251 | 20 | 0.73 (0.63–0.84) |
| **Penn-Nicholson6** | 258 | 20 | 0.69 (0.59–0.78) |
| **Rajan5** | 215 | 12 | 0.54 (0.35–0.73) |
| **Roe1** | 258 | 20 | 0.53 (0.41–0.65) |
| **Roe3** | 257 | 20 | 0.53 (0.37–0.68) |
| **Sambarey10** | 254 | 20 | 0.69 (0.61–0.77) |
| **Satproedprai7** | 254 | 20 | 0.70 (0.60–0.81) |
| **Suliman2** | 149 | 4 | 0.54 (0.29–0.79) |
| **Suliman4** | 249 | 20 | 0.62 (0.49–0.75) |
| **Sweeney3** | 248 | 20 | 0.69 (0.55–0.82) |
| **Thompson5** | 257 | 19 | 0.82 (0.73–0.90) |
| **Xpert HR** | 259 | 20 | 0.52 (0.37–0.67) |

AUC=area under the curve. CI=confidence interval. Xpert HR=Xpert Host-Response.

## Table S8B. Sensitivity analysis: Performance of transcriptomic signatures measured at baseline, versus covariate-adjusted signature models, for predicting death due to TB or unknown cause.

| **Signature** | **Baseline covariate-adjusted model**  **ΔAUC (95% CI; p-value)** |
| --- | --- |
| **da Costa3** | **0.21 (0.06–0.36; 0.0056)** |
| **de Araujo1** | **0.19 (0.04–0.34; 0.014)** |
| **Duffy4** | 0.03 (-0.02–0.08; 0.22) |
| **Duffy9** | **0.09 (0.00–0.18; 0.049)** |
| **Francisco2** | **0.17 (0.02–0.32; 0.026)** |
| **Gjoen7** | **0.20 (0.05–0.35; 0.0077)** |
| **Gliddon3** | **0.09 (0.01–0.16; 0.029)** |
| **Gliddon4** | 0.04 (-0.03–0.10; 0.24) |
| **Jacobsen3** | 0.09 (0.00–0.18; 0.057) |
| **Kaforou22** | 0.07 (-0.01–0.15; 0.11) |
| **Maertzdorf4** | 0.06 (-0.01–0.14; 0.10) |
| **Penn-Nicholson6** | **0.12 (0.03–0.21; 0.011)** |
| **Rajan5** | 0.10 (-0.08–0.27; 0.28) |
| **Roe1** | **0.20 (0.07–0.33; 0.0022)** |
| **Roe3** | **0.20 (0.02–0.37; 0.026)** |
| **Sambarey10** | **0.13 (0.06–0.21; 0.0006)** |
| **Satproedprai7** | **0.11 (0.02–0.19; 0.017)** |
| **Suliman2** | 0.16 (-0.17–0.49; 0.33) |
| **Suliman4** | **0.14 (0.01–0.28; 0.031)** |
| **Sweeney3** | 0.08 (0.00–0.17; 0.056) |
| **Thompson5** | 0.04 (0.00–0.09; 0.053) |
| **Xpert HR** | **0.21 (0.04–0.39; 0.019)** |

AUC=area under the curve. CI=confidence interval. Xpert HR=Xpert Host-Response.

ΔAUC is the difference in AUC between the covariate-adjusted signature model (signature + age + sex + HIV) minus the AUC for the base signature model (signature only).

## Table S9. Sensitivity, specificity, positive predictive value, and negative predictive value of transcriptomic signatures measured at baseline for predicting death due to TB or unknown cause.

| **Signature** | **Baseline sensitivity, %**  **(95% CI)** | **Baseline specificity, %**  **(95% CI)** |
| --- | --- | --- |
| **WHO Target Product Profile for TB treatment biomarkers** | Minimum: 90%  Optimum: 95% | **Minimum: 70% ***  Optimum: 80% |
| **da Costa3** | 40.0 (21.9–61.3) | 69.9 (64.0–75.1) |
| **de Araujo1** | 45.0 (25.8–65.8) | 69.9 (64.0–75.1) |
| **Duffy4** | 78.9 (56.7–91.5) | 69.5 (63.5–74.9) |
| **Duffy9** | 57.9 (36.3–76.9) | 69.9 (63.9–75.2) |
| **Francisco2** | 35.0 (18.1–56.7) | 70.6 (64.6–75.9) |
| **Gjoen7** | 40.0 (21.9–61.3) | 69.9 (64.0–75.1) |
| **Gliddon3** | 52.9 (31.0–73.8) | 69.5 (63.5–75.0) |
| **Gliddon4** | 66.7 (43.7–83.7) | 69.7 (63.8–75.0) |
| **Jacobsen3** | 53.8 (29.1–76.8) | 69.6 (63.2–75.3) |
| **Kaforou22** | 62.5 (38.6–81.5) | 69.6 (63.3–75.2) |
| **Maertzdorf4** | 65.0 (43.3–81.9) | 69.7 (63.8–75.1) |
| **Penn-Nicholson6** | 40.0 (21.9–61.3) | 69.8 (63.9–75.0) |
| **Rajan5** | 41.7 (19.3–68.0) | 69.8 (63.3–75.5) |
| **Roe1** | 30.0 (14.5–51.9) | 69.8 (63.9–75.0) |
| **Roe3** | 45.0 (25.8–65.8) | 69.6 (63.8–74.9) |
| **Sambarey10** | 35.0 (18.1–56.7) | 69.7 (63.8–75.0) |
| **Satproedprai7** | 60.0 (38.7–78.1) | 69.7 (63.8–75.0) |
| **Suliman2** | 25.0 ( 4.6–69.9) | 69.8 (62.0–76.6) |
| **Suliman4** | 40.0 (21.9–61.3) | 69.9 (63.9–75.2) |
| **Sweeney3** | 70.0 (48.1–85.5) | 69.8 (63.8–75.1) |
| **Thompson5** | 84.2 (62.4–94.5) | 69.6 (63.8–74.9) |
| **Xpert HR** | 45.0 (25.8–65.8) | 69.9 (64.0–75.1) |

* Signatures benchmarked at 70% specificity.

CI=confidence interval. Xpert HR=Xpert Host-Response.

## Table S10. Multivariable linear regression analysis to identify factors associated with baseline Thompson5 and XpertHR signature scores.

|  | **Thompson5** | | **Xpert HR** | |
| --- | --- | --- | --- | --- |
|  | **β coefficient**  **(95% CI)** | **p-value** | **β coefficient**  **(95% CI)** | **p-value** |
| (Intercept) | 0.75 (0.52; 0.97) | <0.001 | 0.83 (0.59; 1.07) | <0.001 |
| Sex |  |  |  |  |
| Female | Reference |  | Reference |  |
| Male | 0.04 (-0.03; 0.10) | 0.25 | 0.01 (-0.05; 0.07) | 0.76 |
| Age (per 10 years) | -0.01 (-0.03; 0.01) | 0.39 | -0.03 (-0.05; -0.01) | **0.003** |
| City |  |  |  |  |
| Manaus | Reference |  | Reference |  |
| Rio de Janeiro | -0.06 (-0.14; 0.03) | 0.22 | 0 (-0.09; 0.09) | >0.99 |
| Salvador | 0 (-0.09; 0.10) | 0.98 | 0.03 (-0.07; 0.13) | 0.49 |
| Ethnicity |  |  |  |  |
| Black | Reference |  | Reference |  |
| Pardo | 0.05 (-0.04; 0.13) | 0.26 | -0.02 (-0.11; 0.07) | 0.63 |
| Indian | 0.17 (-0.05; 0.38) | 0.13 | 0.12 (-0.11; 0.35) | 0.31 |
| White | 0.04 (-0.05; 0.14) | 0.37 | -0.08 (-0.19; 0.02) | 0.11 |
| Unknown | -0.12 (-0.57; 0.33) | 0.60 | -0.13 (-0.60; 0.34) | 0.59 |
| Smoking history |  |  |  |  |
| Never smoked | Reference |  | Reference |  |
| Former smoker | -0.01 (-0.06; 0.04) | 0.64 | -0.01 (-0.06; 0.04) | 0.57 |
| Current smoker | 0.05 (-0.01; 0.11) | 0.13 | 0.04 (-0.03; 0.10) | 0.28 |
| Baseline body-mass index (per 10 kg/m^2^) | -0.01 (-0.02; 0) | 0.087 | 0 (-0.01; 0.010) | 0.45 |
| Baseline HIV status |  |  |  |  |
| Negative | Reference |  | Reference |  |
| Positive | -0.04 (-0.12; 0.03) | 0.25 | -0.04 (-0.12; 0.04) | 0.33 |
| Sputum smear status |  |  |  |  |
| Scanty | Reference |  | Reference |  |
| 1+ | 0.06 (-0.03; 0.15) | 0.19 | 0.07 (-0.02; 0.17) | 0.13 |
| 2+ | 0.05 (-0.05; 0.14) | 0.35 | **0.11 (0.01; 0.21)** | **0.038** |
| 3+ | 0.10 (0; 0.20) | **0.040** | **0.15 (0.05; 0.25)** | **0.004** |
| Chest radiographic evidence of cavitation |  |  |  |  |
| No | Reference |  | Reference |  |
| Yes | 0.04 (-0.03; 0.11) | 0.26 | -0.04 (-0.11; 0.03) | 0.30 |
| Uncertain | 0.05 (-0.06; 0.16) | 0.40 | 0 (-0.12; 0.11) | 0.99 |
| TB disease outcome |  |  |  |  |
| Recurrence-free cure | Reference |  | Reference |  |
| Death due to TB or unknown causes | **0.18 (0.07; 0.29)** | **0.002** | 0.04 (-0.08; 0.16) | 0.51 |
| Treatment failure | **0.09 (0; 0.19)** | **0.045** | 0.04 (-0.06; 0.13) | 0.43 |
| TB recurrence | **0.23 (0.06; 0.40)** | **0.007** | **0.23 (0.05; 0.40)** | **0.014** |
| **Summary Statistics** |  |  |  |  |
| Adjusted R^2^ | 0.12 |  | 0.07 |  |
| Missing variables | 72 |  | 73 |  |
| Degrees of freedom | 227 |  | 230 |  |

CI=confidence interval. Xpert HR=Xpert Host-Response

# Supplemental References

1.	Glynn RJ, Gagne JJ, Schneeweiss S. Role of disease risk scores in comparative effectiveness research with emerging therapies. Pharmacoepidemiol Drug Saf. 2012;21 Suppl 2(Suppl 2):138-47. doi: 10.1002/pds.3231.

2.	Muwanga VM, Mendelsohn SC, Leukes V, Stanley K, Mbandi SK, Erasmus M, et al. Blood transcriptomic signatures for symptomatic tuberculosis in an African multicohort study. European Respiratory Journal. 2024;64(2). doi: 10.1183/13993003.00153-2024.

3.	Mendelsohn SC, Andrade BB, Mbandi SK, Andrade AMS, Muwanga VM, Figueiredo MC, et al. Transcriptomic Signatures of Progression to Tuberculosis Disease Among Close Contacts in Brazil. Journal of Infectious Diseases. 2024;230(6):e1355-e65. doi: 10.1093/infdis/jiae237.

4.	da Costa LL, Delcroix M, Costa ERD, Prestes IV, Milano M, Francis SS, et al. A real-time PCR signature to discriminate between tuberculosis and other pulmonary diseases. Tuberculosis. 2015;95(4):421-5. doi: 10.1016/j.tube.2015.04.008.

5.	Duffy FJ, Thompson EG, Scriba TJ, Zak DE. Multinomial modelling of TB/HIV co-infection yields a robust predictive signature and generates hypotheses about the HIV+TB+ disease state. PLoS ONE. 2019;14(7):e0219322. doi: 10.1371/journal.pone.0219322.

6.	Francisco NM, Fang YM, Ding L, Feng S, Yang Y, Wu M, et al. Diagnostic accuracy of a selected signature gene set that discriminates active pulmonary tuberculosis and other pulmonary diseases. Journal of Infection. 2017;75(6):499-510. doi: 10.1016/j.jinf.2017.09.012.

7.	Gjoen JE, Jenum S, Sivakumaran D, Mukherjee A, Macaden R, Kabra SK, et al. Novel transcriptional signatures for sputum-independent diagnostics of tuberculosis in children. Sci Rep. 2017;7(1):5839. doi: 10.1038/s41598-017-05057-x.

8.	Jacobsen M, Repsilber D, Gutschmidt A, Neher A, Feldmann K, Mollenkopf HJ, et al. Candidate biomarkers for discrimination between infection and disease caused by Mycobacterium tuberculosis. J Mol Med (Berl). 2007;85(6):613-21. doi: 10.1007/s00109-007-0157-6.

9.	Roe J, Venturini C, Gupta RK, Gurry C, Chain BM, Sun Y, et al. Blood Transcriptomic Stratification of Short-term Risk in Contacts of Tuberculosis. Clinical Infectious Diseases. 2020;70(5):731-7. doi: 10.1093/cid/ciz252.

10.	Sambarey A, Devaprasad A, Mohan A, Ahmed A, Nayak S, Swaminathan S, et al. Unbiased Identification of Blood-based Biomarkers for Pulmonary Tuberculosis by Modeling and Mining Molecular Interaction Networks. EBioMedicine. 2017;15:112-26. doi: 10.1016/j.ebiom.2016.12.009.

11.	Satproedprai N, Wichukchinda N, Suphankong S, Inunchot W, Kuntima T, Kumpeerasart S, et al. Diagnostic value of blood gene expression signatures in active tuberculosis in Thais: a pilot study. Genes and Immunity. 2015;16(4):253-60. doi: 10.1038/gene.2015.4.

12.	Darboe F, Mbandi SK, Naidoo K, Yende-Zuma N, Lewis L, Thompson EG, et al. Detection of Tuberculosis Recurrence, Diagnosis and Treatment Response by a Blood Transcriptomic Risk Signature in HIV-Infected Persons on Antiretroviral Therapy. Front Microbiol. 2019;10:1441. doi: 10.3389/fmicb.2019.01441.

13.	Penn-Nicholson A, Mbandi SK, Thompson E, Mendelsohn SC, Suliman S, Chegou NN, et al. RISK6, a 6-gene transcriptomic signature of TB disease risk, diagnosis and treatment response. Sci Rep. 2020;10(1):8629. doi: 10.1038/s41598-020-65043-8.

14.	Gliddon HD, Kaforou M, Alikian M, Habgood-Coote D, Zhou C, Oni T, et al. Identification of Reduced Host Transcriptomic Signatures for Tuberculosis Disease and Digital PCR-Based Validation and Quantification. Front Immunol. 2021;12:637164. doi: 10.3389/fimmu.2021.637164.

15.	Kaforou M, Wright VJ, Oni T, French N, Anderson ST, Bangani N, et al. Detection of tuberculosis in HIV-infected and -uninfected African adults using whole blood RNA expression signatures: a case-control study. PLoS Medicine. 2013;10(10):e1001538. doi: 10.1371/journal.pmed.1001538.

16.	Rajan JV, Semitala FC, Mehta T, Seielstad M, Montalvo L, Andama A, et al. A Novel, 5-Transcript, Whole-blood Gene-expression Signature for Tuberculosis Screening Among People Living With Human Immunodeficiency Virus. Clinical Infectious Diseases. 2019;69(1):77-83. doi: 10.1093/cid/ciy835.

17.	Stopsack K, Gerke T. batchtma: Batch Effect Adjustments. 2021. <https://CRAN.R-project.org/package=batchtma>.

18.	Signorell A, Aho K, Alfons A, Anderegg N, Aragon T, Arppe A, et al. DescTools: Tools for descriptive statistics. 2019. <https://cran.r-project.org/web/packages/DescTools/index.html>.

19.	de Araujo LS, Vaas LA, Ribeiro-Alves M, Geffers R, Mello FC, de Almeida AS, et al. Transcriptomic Biomarkers for Tuberculosis: Evaluation of DOCK9. EPHA4, and NPC2 mRNA Expression in Peripheral Blood. Front Microbiol. 2016;7:1586. doi: 10.3389/fmicb.2016.01586.

20.	Duffy FJ, Olson GS, Gold ES, Jahn A, Aderem A, Aitchison JD, et al. Use of a Contained Mycobacterium tuberculosis Mouse Infection Model to Predict Active Disease and Containment in Humans. Journal of Infectious Diseases. 2022;225(10):1832-40. doi: 10.1093/infdis/jiab130.

21.	Maertzdorf J, McEwen G, Weiner J, 3rd, Tian S, Lader E, Schriek U, et al. Concise gene signature for point-of-care classification of tuberculosis. EMBO Molecular Medicine. 2016;8(2):86-95. doi: 10.15252/emmm.201505790.

22.	Roe JK, Thomas N, Gil E, Best K, Tsaliki E, Morris-Jones S, et al. Blood transcriptomic diagnosis of pulmonary and extrapulmonary tuberculosis. JCI Insight. 2016;1(16):e87238. doi: 10.1172/jci.insight.87238.

23.	Suliman S, Thompson EG, Sutherland J, Weiner J, 3rd, Ota MOC, Shankar S, et al. Four-Gene Pan-African Blood Signature Predicts Progression to Tuberculosis. American Journal of Respiratory and Critical Care Medicine. 2018;197(9):1198-208. doi: 10.1164/rccm.201711-2340OC.

24.	Warsinske HC, Rao AM, Moreira FMF, Santos PCP, Liu AB, Scott M, et al. Assessment of Validity of a Blood-Based 3-Gene Signature Score for Progression and Diagnosis of Tuberculosis, Disease Severity, and Treatment Response. JAMA Netw Open. 2018;1(6):e183779. doi: 10.1001/jamanetworkopen.2018.3779.

25.	Sweeney TE, Braviak L, Tato CM, Khatri P. Genome-wide expression for diagnosis of pulmonary tuberculosis: a multicohort analysis. Lancet Respir Med. 2016;4(3):213-24. doi: 10.1016/S2213-2600(16)00048-5.

26.	Thompson EG, Du Y, Malherbe ST, Shankar S, Braun J, Valvo J, et al. Host blood RNA signatures predict the outcome of tuberculosis treatment. Tuberculosis (Edinb). 2017;107:48-58. doi: 10.1016/j.tube.2017.08.004.

27.	Gupta-Wright A, Ha H, Abdulgadar S, Crowder R, Emmanuel J, Mukwatamundu J, et al. Evaluation of the Xpert MTB Host Response assay for the triage of patients with presumed pulmonary tuberculosis: a prospective diagnostic accuracy study in Viet Nam, India, the Philippines, Uganda, and South Africa. Lancet glob health. 2024;12(2):e226-e34. doi: 10.1016/S2214-109X(23)00541-7.

28.	Gupta-Wright A, den Boon S, MacLean E, Cirillo D, Cobelens F, Gillespie S, et al. Target product profiles: tests for tuberculosis treatment monitoring and optimization. Bulletin of the World Health Organization. 2023;101(11):730-7. doi: 10.2471/blt.23.290901.

29.	WHO. Target product profiles for tests for tuberculosis treatment monitoring and optimization. Geneva: World Health Organization, 2023. <https://www.who.int/publications/i/item/9789240081178> (accessed 22 March 2024).
